# The Complete Chloroplast Genome Sequences of Five *Epimedium* Species: Lights into Phylogenetic and Taxonomic Analyses

**DOI:** 10.3389/fpls.2016.00306

**Published:** 2016-03-15

**Authors:** Yanjun Zhang, Liuwen Du, Ao Liu, Jianjun Chen, Li Wu, Weiming Hu, Wei Zhang, Kyunghee Kim, Sang-Choon Lee, Tae-Jin Yang, Ying Wang

**Affiliations:** ^1^Key Laboratory of Plant Germplasm Enhancement and Specialty Agriculture, Wuhan Botanical Garden, Chinese Academy of SciencesWuhan, China; ^2^College of Life Science, University of Chinese Academy of SciencesBeijing, China; ^3^College of Life Sciences, Xinyang Normal UniversityXinyang, China; ^4^Department of Plant Science, College of Agriculture and Life Sciences, Plant Genomics and Breeding Institute, and Research Institute of Agriculture and Life Sciences, Seoul National UniversitySeoul, South Korea; ^5^Key Laboratory of South China Agricultural Plant Molecular Analysis and Genetic Improvement, Provincial Key Laboratory of Applied Botany, South China Botanical Garden, Chinese Academy of SciencesGuangzhou, China

**Keywords:** *Epimedium*, chloroplast genome, genome structure, phylogenetic relationships, taxonomic identification

## Abstract

*Epimedium* L. is a phylogenetically and economically important genus in the family Berberidaceae. We here sequenced the complete chloroplast (cp) genomes of four *Epimedium* species using Illumina sequencing technology via a combination of *de novo* and reference-guided assembly, which was also the first comprehensive cp genome analysis on *Epimedium* combining the cp genome sequence of *E. koreanum* previously reported. The five *Epimedium* cp genomes exhibited typical quadripartite and circular structure that was rather conserved in genomic structure and the synteny of gene order. However, these cp genomes presented obvious variations at the boundaries of the four regions because of the expansion and contraction of the inverted repeat (IR) region and the single-copy (SC) boundary regions. The *trnQ*-*UUG* duplication occurred in the five *Epimedium* cp genomes, which was not found in the other basal eudicotyledons. The rapidly evolving cp genome regions were detected among the five cp genomes, as well as the difference of simple sequence repeats (SSR) and repeat sequence were identified. Phylogenetic relationships among the five *Epimedium* species based on their cp genomes showed accordance with the updated system of the genus on the whole, but reminded that the evolutionary relationships and the divisions of the genus need further investigation applying more evidences. The availability of these cp genomes provided valuable genetic information for accurately identifying species, taxonomy and phylogenetic resolution and evolution of *Epimedium*, and assist in exploration and utilization of *Epimedium* plants.

## Introduction

*Epimedium* comprising about 58 species, is a phylogenetically and economically important genus in the family Berberidaceae (Stearn, 2002; Ying et al., 2011). As the diversity center of *Epimedium*, China possesses about 48 species, and has used *Epimedium* plants as herb-medicine for more than 2000 years. Herb epimedii has been verified with activity in nourishing the kidney, reinforcing the Yang, regulating bone remodeling, curing cardiovascular diseases, possessing anti-cancer, and anti-aging benefits (Ma et al., 2011; Jiang et al., 2015). The kind and quantity of drugs and health products with herb epimedii as raw materials have been increasing in the last 20 years, which has led to substantial appreciation of prices of the medicinal materials. Furthermore, bearing attractive foliage and flowers, *Epimedium* plants were previously mainly introduced as perennial garden plant in Europe and America. At present, the horticultural values of *Epimedium* plants have been widely paid attention with great commercial prospects (Lubell and Brand, 2005; Ren et al., 2008; Avent, 2010).

*Epimedium* is taxonomically and phylogenetically regarded as one of the most challengingly difficult taxa in plants. The updated system of *Epimedium* classified the genus into two subgenera, four sections, and four series mainly based on geographical distribution, and leaf, and flower morphology (Stearn, 2002). However, molecular phylogenetic analyses based on internal transcribed spacer (ITS), *trnK*-*matK, atpB*-*rbcL* spacer sequences, and amplified fragment length polymorphisms (AFLPs) only consistently supported subg. *Rhizophyllum* and four sections of subg. *Epimedium* as five distinctive clades (Sun et al., 2005; Zhang et al., 2007, 2014; De Smet et al., 2012). The two subgenera were not well-supported, the relationships between five clades were unresolved except for sect. *Epimedium* as sister to sect. *Macroceras*, as well as the four series of sect. *Diphyllon* being poorly supported. As a genus of basal eudicots in North Temperate Zone, the five clades of *Epimedium* have their unique distribution regions, respectively, and with enormous gaps. It needs more effective molecular markers to investigate the relationships between the five clades and classification system of *Epimedium*, as well as the origin, evolution, migration, and dispersal of the genus in North Temperate Zone.

It has been intractable for the species identification of *Epimedium*, particularly for those of sect. *Diphyllon*, which baffled the effective exploration and utilization of the genus. Chinese sect. *Diphyllon* has highest species diversity level with about 47 species, and sympatric distribution, and hybridization made the interspecies relationship very complicated. Furthermore, many species, such as *E. sagittatum, E. pubescens*, and *E. acuminatum*, have abundant infra-species variations in morphology and medicinal ingredients. However, only AFLPs were heretofore and successfully applied to identify the species of sect. *Diphyllon* (Zhang et al., 2014). Internal primer binding sites (iPBS) were used to investigate the intra-species variations of *E. sagittatum* (Chen et al., 2015). For conservation, utilization, and domestication of *Epimedium* plants, more effective molecular markers are needed to identify *Epimedium* species and conduct the population genetics and breeding for the *Epimedium* genus.

The chloroplast (cp) is an important plastid that plays a key role in plant cell for photosynthesis and carbon fixation (Neuhaus and Emes, 2000). The cp genomes in angiosperms are circular DNA molecules ranging from 115 to 165 kb in length and consisting of two copies of a large inverted repeat (IR) region separated by a large-single-copy (LSC) region and a small-single-copy (SSC) region (Raubeson and Jansen, 2005; Wicke et al., 2011). The cp genomes could provide valuable information for taxonomy and phylogeny as a result of sequence divergence between plant species and individuals (Jansen et al., 2007; Moore et al., 2007; Parks et al., 2009; Huang et al., 2014; Jung et al., 2014). Owing to being haploid, maternal inheritance, and high conservation in gene content and genome structure, the cp genomes have been popular to study the evolutionary relationships at almost any taxonomic level in plants. With the advent of high-throughput sequencing technologies, it is now more practical and inexpensive to obtain cp genome sequences and promote cp-based phylogenetics to phylogenomics.

In this study, we sequenced the cp genomes of four *Epimedium* species using the next-generation sequencing platform, which is also the first comprehensive analysis on cp genomes for *Epimedium* combining the cp genome of *E. koreanum* previously reported (Lee et al., 2015). Our study aims were as follows: (1) to investigate global structural patterns of *Epimedium* cp genomes; (2) to screen sequence divergence hotspot regions in the five *Epimedium* cp genomes; (3) to examine variations of simple sequence repeats (SSRs) and repeat sequences among the five *Epimedium* cp genomes; (4) to reconstruct phylogenetic relationships among the five *Epimedium* species using their cp genome sequences. The results will provide abundant information for further species identification, taxonomy and phylogenetic resolution of *Epimedium*, and assist in exploration and utilization of *Epimedium* plants.

## Materials and methods

### Sample preparation, sequencing, assembly, and validation

Fresh leaves of five *Epimedium* species, four from China, and one from Korea, were sampled. The samples of four Chinese species were used for complete cp genome sequencing, while that of *E. koreanum* from Korea was only used for PCR-based validating its cp genome sequence (KM207675) previously reported (Lee et al., 2015). The voucher herbarium specimens of four Chinese species were deposited at the Herbaria of Wuhan Botanical Garden, Chinese Academy of Sciences (HIB), and the sample of *E. koreanum* was deposited at Wuhan Botanical Garden, Chinese Academy of Sciences, Hallym University and Seoul National University (Table S1). Total genomic DNA per species was extracted from 100 mg fresh leaves using the DNeasy Plant MiniKit (Qiagen, CA, USA).

For the four Chinese *Epimedium* species, Purified DNA (5 mg) was sheared by nebulization with compressed nitrogen gas, yielding fragments of 300 bp in length, and fragmentation quality was checked on a Bioanalyzer 2100 (Agilent Technologies). Paired-end libraries were constructed following the manufacturer's protocol (Illumina, San Diego, California, USA). Genomic DNAs of four species were sequenced on a single lane on HiSeq2000 flow cell lanes (Illumina Inc.) by National Instrumentation Center for Environmental Management (NICEM; http://nature.snu.ac.kr/kr.php), Seoul, Korea.

For each of the four Chinese *Epimedium* species, cp genome reads were extracted by mapping all raw reads to the reference cp genome of *Nandina domestica* (DQ923117) with BWA (Li and Durbin, 2009). High quality reads were obtained using the CLC-quality trim tool with Phred scores of < 20 and assembled using the CLC genome assembler v4.06 (http://www.clcbio.com/products/clc-assembly-cell) with default parameters. Sequence gaps were filled by Gapcloser included in the SOAP package v1.12 (Li et al., 2010). All the contigs were aligned to the reference cp genome of *Nandina domestica* using MUMmer (Kurtz et al., 2004), and aligned contigs were ordered according to the reference cp genome. Based on the reference cp genome, the four junctions between LSC/IRs and SSC/IRs of the five sampled *Epimedium* species were validated with PCR-based conventional Sanger sequencing, respectively. To avoid assembly errors and obtain high quality complete cp genome sequences, validation of assembly was also carried out on 10 chloroplast genes (Table S2).

### Genome annotation and analysis

Initial gene annotation of the five chloroplast genomes (including that of *E. koreanum*, KM207675) was performed with Dual Organellar GenoMe Annotator (DOGMA; Wyman et al., 2004). DOGMA annotations were manually corrected for the start and stop codons and intron/exon boundaries by comparison to homologous genes from other sequenced cp genomes in Ranales. The tRNA genes were also verified with ARAGORN (Laslett and Canback, 2004) and tRNAscan-SE (Lowe and Eddy, 1997; Schattner et al., 2005). The circular cp genome maps were drawn using the OrganellarGenome DRAW tool (ORDRAW; Lohse et al., 2007), with subsequent manual editing.

Cp genome comparison among the five *Epimedium* species was performed with the mVISTA program (Frazer et al., 2004). Genome, protein coding gene, intron, and spacer sequence divergences were evaluated using DnaSP 5.10 (Rozas et al., 2003) after aligned. The genome sequences were aligned using MAFFT v5 (Katoh and Toh, 2010) and adjusted manually where necessary. For the protein coding gene sequences, introns and spacers, every gene or fragment was edited using ClustalW multiple alignment option within the software BioEdit v7.0.9.0 (Hall, 2011).

Microsatellites (mono-, di-, tri-, tetra-, penta-, and hexanucleotide repeats) were detected using the Perl script MISA (Thiel et al., 2003) with thresholds of ten repeat units for mononucleotide SSRs, five repeat units for di- and trinucleotide SSRs, and three repeat units for tetra-, penta-, and hexanucleotide SSRs. Size and location of both direct (forward) and inverted (palindromic) repeats in the *Epimedium* cp genome were identified by running REPuter (Kurtz et al., 2001) according to the following criteria: cutoff n ≥30% bp and 90% sequence identities (Hamming distance of 3).

### Phylogenetic analysis

It was found that *trnQ-UUG* genes were duplicated in the LSC of the five *Epimedium* cp genomes, which was not found in other basal eudicotyledons. For investigating the evolution of *trnQ-UUG* gene of *Epimedium*, phylogenetic analyses was conducted based on the nucleotide sequence of the gene of *Epimedium* and other taxa of basal eudicots. The phylogenetic analyses were also performed for the five *Epimedium* species with *Nandina domestica* and *Aconitum barbatum* of Ranales as outgroups. The analyses were carried out based on the following three data sets: (1) the complete cp DNA sequences; (2) protein coding sequences; (3) the introns and spacers. The nucleotide sequence data of *trnQ-UUG* gene and cp genome, except those of the four Chinese *Epimedium* species, were obtained from NCBI, which the sequence data of *trnQ-UUG* gene were also obtained from the corresponding Genbank files of cp genome sequence data (Table S3).

Maximum parsimony (MP) analyses were conducted using PAUP v4b10 (Swofford, 2003). Heuristic search were performed with 1000 random addition sequences, 10 trees held at each step, tree-bisection-reconnection (TBR) branch swapping and MulTrees switched off. Branch support was assessed with 1000 bootstrap replicates with 10 random taxon additions each and TBR and MulTtrees ON. Maximum likelihood (ML) analyses were performed using RAxML-HPC BlackBox v.8.1.24 on the CIPRES Science Gateway website (Stamatakis et al., 2008; Miller et al., 2010). The best-fitting model was selected using ModelTest v.0.1.1 (Posada, 2008), and branch support was estimated with 1000 bootstrap replicates.

## Results and discussions

### Genome assembly and PCR-Based validation

Using the Illumina HiSeq 2000 system, five *Epimedium* species were sequenced to produce 4,573,881–4,675,703 paired-end raw reads (101 bp in average reads length). After screening these paired-end reads through alignment with reference cp genomes of *Nandina domestica*, 84,589 to 236,730 cp genome reads were extracted with 50 × to 145 × coverage (Table 1). Four junction regions and 10 cp genes were validated by PCR-based sequencing in each of the five *Epimedium* cp genomes. The PCR-based sequencing on *E. koreanum* demonstrated identical with its original *de novo* assembly of complete cp genome sequence (KM20267; Lee et al., 2015). However, some initial gene annotations on the sequence were inaccurate, for example that only one *trnQ-UUG* was identified while two copies of *trnQ-UUG* were actually located in LSC. We hereon updated the annotation on the cp genome sequence of *E. koreanum* with Genbank accession number KU522471. The four Chinese *Epimedium* cp genome sequences were also deposited in GenBank (accession numbers, KU522469, KU522470, KU522472, and KU522473).

**Table 1:** **Summary of the sequencing data for five ***Epimedium*** species**.

**Species**	**Raw read no**.	**Total read length (bp)**	**Mapped read no**.	**Mapped to reference genome (%)**	**Cp genome coverage (×)**	**Cp genome length (bp)**	**LSC length (bp)**	**SSC length (bp)**	**IR length (bp)**	**GC content (%)**
*E. acuminatum*	4,638,760	468,514,760	84,589	1.82	49.78	159,112	86,561	17,069	27,741	38.81
*E. dolichostemon*	4,622,454	466,867,854	138,527	3.00	84.83	157,039	88,394	17,077	25,784	38.80
*E. lishihchenii*	4,675,703	472,246,003	99,587	2.13	60.12	157,692	88,420	16,094	26,589	38.77
*E. pseudowushanense*	4,573,881	461,961,981	131,895	2.88	80.24	157,168	88,531	17,069	25,784	38.77
*E. koreanum*	4,612,264	465,838,664	236,730	5.10	144.73	157,218	89,560	17,222	25,218	38.72

### Genome features

**Figure 1:**
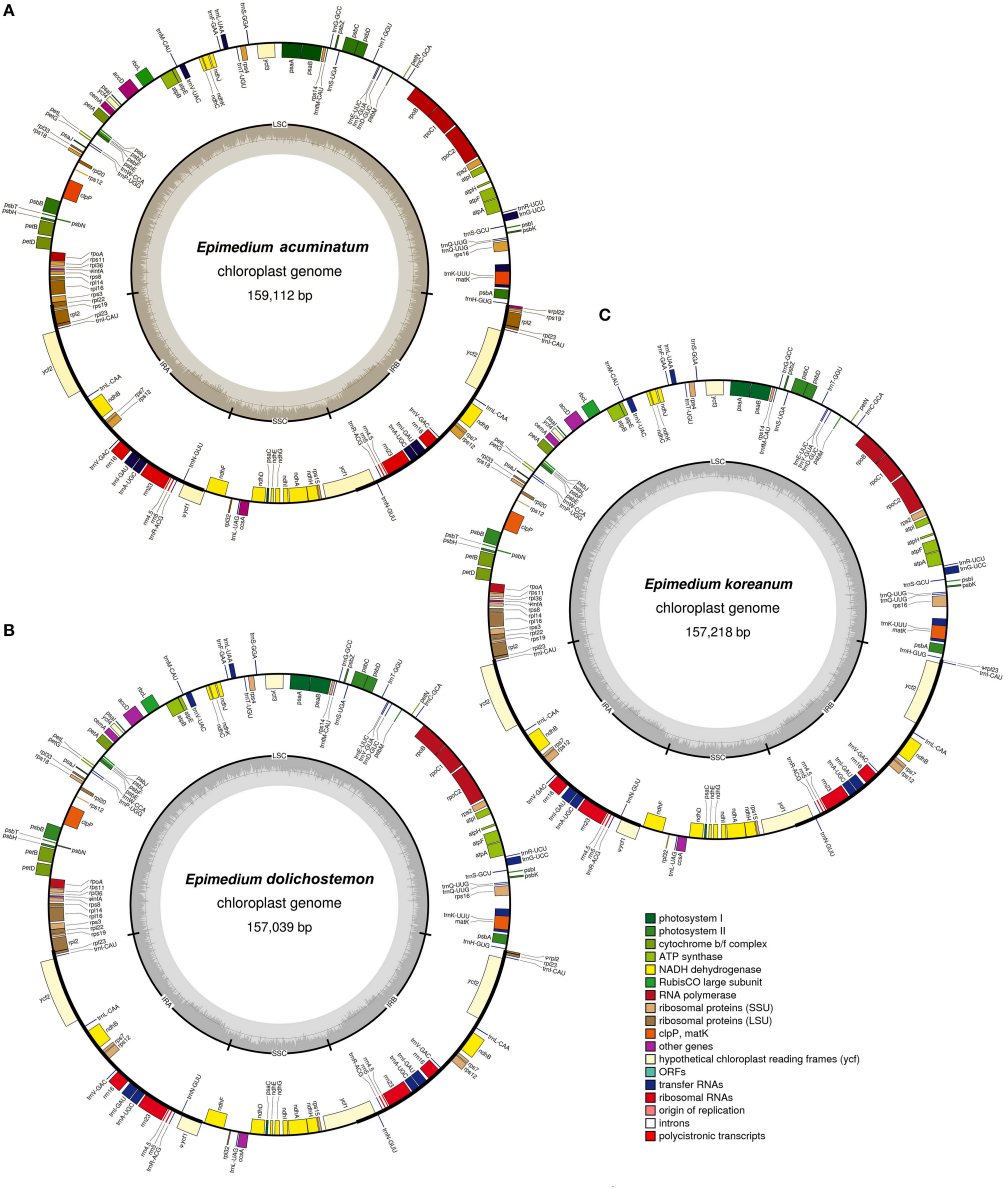
**Gene maps of three ***Epimedium***chloroplast genomes. (A)** *E. acuminatum*, **(B)**. *E. dolichostemon*, **(C)**. *E. koreanum*. Genes shown outside the outer circle are transcribed clockwise, and those inside are transcribed counterclockwise. Genes belonging to different functional groups are color coded. The dashed area in the inner circle indicates GC content of the chloroplast genomes.

The nucleotide sequences of the five *Epimedium* cp genomes ranged from 157,039 bp (*E. acuminatum*) to 159,112 bp (*E. dolichostemon*; Figure 1, Table 1). All the five cp genomes displayed the typical quadripartite structure of angiosperms, which consisted of a pair of IR regions (25,218–27,741 bp) separated by a LSC region (86,561–89,560 bp), and a SSC region (16,094–17,222 bp). The average GC content was ~38.77%, which is almost identical with each other among the five complete *Epimedium* cp genomes.

When duplicated genes in IR regions were counted only once, the five *Epimedium* cp genomes identically harbored 112 different genes arranged in the same order, including 78 protein-coding genes, 30 tRNA, and 4 rRNA. Twelve of the protein-coding genes and six of the tRNA genes contain introns, 15 of which contained a single intron, whereas, three have two introns (Table 2). Among 78 protein-coding genes, 75 genes had the standard AUG as the initiator codon, but *rps14* and *rps19* started with GUG while *rpl2* and *ndhD* with ACG. An ACG codon may be restored to a canonical start codon (AUG) by RNA editing (Hoch et al., 1991; Takenaka et al., 2013), whereas, a GUG initiation codon has been reported in other cp genomes (Kuroda et al., 2007; Gao et al., 2009).

**Table 2:** **List of genes encoded by five ***Epimedium*** chloroplast genome**.

**Category for genes**	**Group of genes**	**Name of genes**
Self-replication	rRNA genes	*rrn16*^a^, *rrn23*^a^, *rrn4.5*^a^, *rrn5*^a^
	tRNA genes	trnA-UGC^*^,^a^, trnC-GCA, trnD-GUC, trnE-UUC, trnF-GAA, trnfM-CAU, trnG-GCC, trnG-UCC^*^, *trnH-GUG, trnI-CAU*^a^, *trnI-GAU*,^a^, trnK-UUU^*^, *trnL-CAA*^a^, trnL-UAA^*^, *trnL-UAG, trnM-CAU, trnN-GUU*^a^, *trnP-UGG, trnQ-UUG*^a^, *trnR-ACG*^a^, *trnR-UCU, trnS-GCU, trnS-GGA, trnS-UGA, trnT-GGU, trnT-UGU, trnV-GAC*^a^, trnV-UAC^*^, *trnW-CCA, trnY-GUA*
	Small subunit of ribosome	*rps2, rps3, rps4, rps7*^a^, rps8, rps11, rps12^**^,^a^, rps14, rps15, rps16^*^, *rps18, rps19*^b^
	Large subunit of ribosome	rpl2^*^,^b^, rpl14, rpl16^*^, *rpl20, rpl22, rpl23*^b^, *rpl32, rpl33, rpl36*
	DNA dependent RNA polymerase	rpoA, rpoB, rpoC1^*^, *rpoC2*
Genes for phytosynthesis	Subunits of NADH-dehydrogenase	ndhA^*^, ndhB^*^,^a^, *ndhC, ndhD, ndhE, ndhF, ndhG, ndhH, ndhI, ndhJ, ndhK*
	Subunits of photosystem I	psaA, psaB, psaC, psaI, psaJ, ycf3^**^
	Subunits of photosystem II	*psbA, psbB, psbC, psbD, psbE, psbF, psbH, psbI, psbJ, psbK, psbL, psbM, psbN, psbT, psbZ*
	Subunits of cytochrome b/f complex	petA, petB^*^, petD^*^, *petG, petL, petN*
	Subunits of ATP synthase	atpA, atpB, atpE, atpF^*^, *atpH, atpI*
	Large subunit of rubisco	*rbcL*
Other genes	Maturase	*matK*
	Protease	clpP^**^
	Envelope membrane protein	*cemA*
	Subunit of Acetyl-CoA-carboxylase	*accD*
	c-type cytochrome synthesis gene	*ccsA*
Genes of unknown function	Open Reading Frames (ORF, ycf)	*ycf1, ycf2*^a^, *ycf4*

* *Gene with one intron*,

** *Gene with two introns*,

a *Gene with two copies*,

b *Gene with one or two copies*.

**Figure 2:**
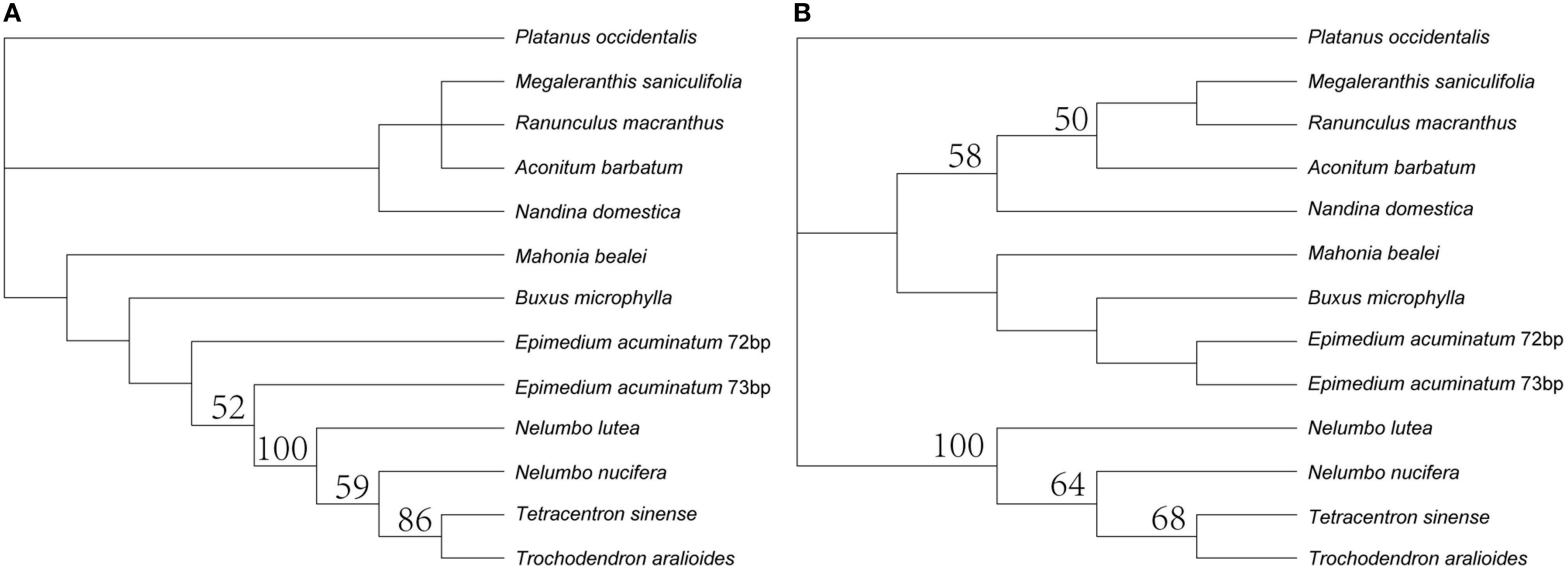
**Phylogenetic trees constructed by ***trnQ-UUG*** sequences of *Epimedium*, and other 11 species of basal eudicotyledons with maximum parsimony (A) and maximum likelihood (B)**. Numbers above node are bootstrap support values (>50%).

The *trnQ-UUG* genes were duplicated in the LSC of the five *Epimedium* cp genomes and coherently separated by 101 bp with the same orientation. The nucleotide sequence of each copy was identical among the five *Epimedium* species. The length of one copy was 72 bp and the other with 73 bp, and the two copies were with 19% sequence divergence. The *trnQ-UUG* duplication had been reported in the family Geraniaceae (Weng et al., 2013), but the gene duplication of *Epimedium* was firstly found in the basal eudicotyledons. Both MP and ML phylogenetic trees based on *trnQ-UUG* sequences of *Epimedium*, and other 11 basal eudicotyledons demonstrated that the two copies of the gene in *Epimedium* had most close relationship (Figure 2). This raised the possibility of independent duplications *of trnQ-UUG* in the genus *Epimedium*.

**Figure 3:**
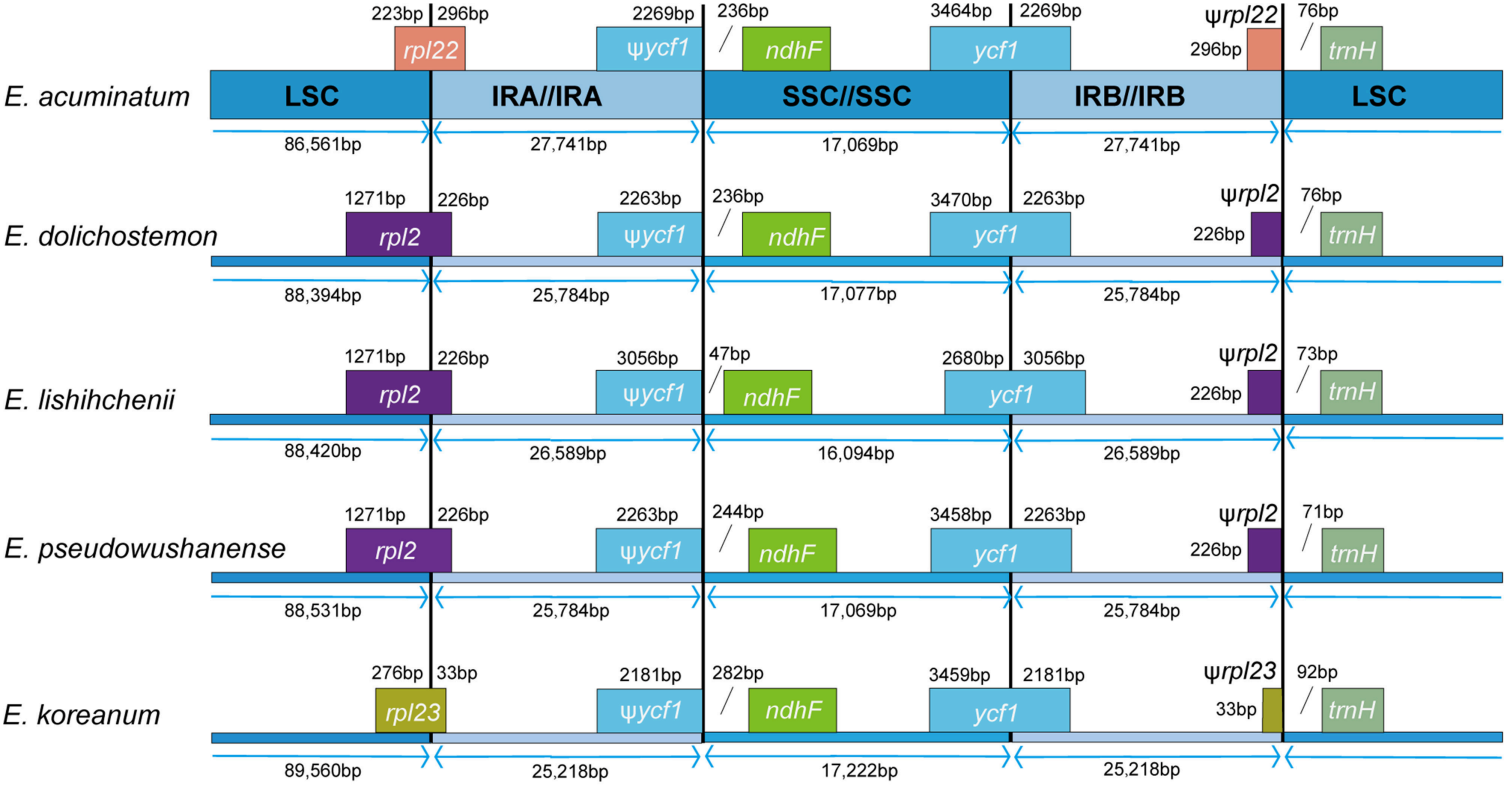
**Comparisons of LSC, SSC, and IR region borders among the five ***Epimedium*** chloroplast genomes**.

The expansion and contraction of the IR region and the single-copy (SC) boundary regions was considered as a primarily mechanism causing the length variation of angiosperm cp genomes (Kim and Lee, 2004). Although overall genomic structure including gene number and gene order were well-conserved, the five *Epimedium* cp genomes exhibited obvious different at the IR/SC boundary regions (Figure 3). The gene *ycf1* crossed the SSC/IRB region, and the pseudogene fragment ψ*ycf1* was located at the IRA region with 2181–3056 bp. The gene *rpl22* crossed the LSC/IRA region in *E. acuminatum*, and ψ*rpl22* with 296 bp was located at IRB region; *rpl2* crossed the LSC/IRA region in *E. dolichostemon, E. lishihchenii*, and *E. pseudowushanense*, and ψ*rpl2* with 226 bp was located at IRB region; *rpl23* crossed the LSC/IRA region in *E. koreanum*, and ψ*rpl23* with 33 bp was located at IRB region. At the junction of IRA/SSC region, the distance between ψ*ycf1* and *ndhF* ranged from 47 to 282 bp. At the junction of IRB/LSC region, the distance between ψ*rpl22* and *trnH* in *E. acuminatum* was 76 bp, the distance between ψ*rpl2* and *trnH* was from 71 to 76 bp in *E. dolichostemon, E. lishihchenii*, and *E. pseudowushanense*, and the distance between ψ*rpl23 and trnH* in *E. koreanum* was 92 bp. The variations at IR/SC boundary regions in the five *Epimedium* cp genomes led to their length variation of the four regions and whole genome sequences.

### Divergence hotspot regions

**Figure 4:**
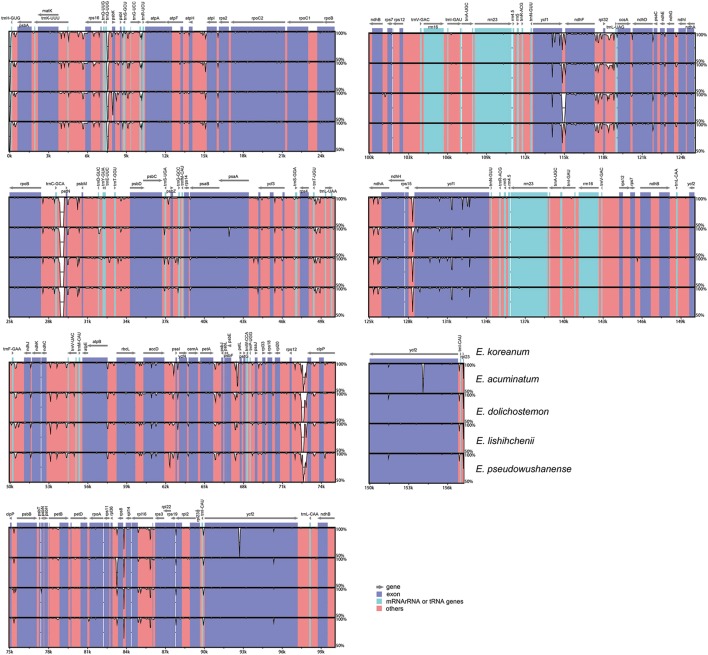
**Sequence identity plots among the five ***Epimedium*** chloroplast genomes**.

For purposes of the subsequent phylogenetic analyses and plant identification, the complete cp genomes of the five *Epimedium* species were compared and plotted using the mVISTA program to elucidate the level of sequence divergence (Figure 4). The IRs had lower sequence divergence than that in the SC regions, which also occurred in most higher plants and possibly due to copy correction between IR sequences by gene conversion (Khakhlova and Bock, 2006). The whole genomes, protein-coding regions (pCDS), and non-coding regions (introns and spacers) exhibited divergence proportions of 3.97%, 1.10%, and 5.81%, respectively. For protein coding regions, *rps16, psbK, rps132, rps14*, and *rps15* had over 3% sequence divergences (Table S4). The non-coding regions had higher variability proportions, and four of the regions at the junction of the IRB and LSC had divergence proportions of 100% because of difference in expansion and contraction of IRB (Table S5). Fifty-two non-coding regions had variability proportions ranging from 3.03 to 86.55%, among which 17 regions, such as *ycf1*/*ndhF, trnC-GCA*/*petN*, and *trnQ-UUG*/*psbK*, had over 10% variability proportions. These divergence hotspot regions of the five *Epimedium* cp genome sequences provided abundant information for developing molecular markers for phylogenetic analyses and plant identification of *Epimedium* species.

### SSR polymorphisms

SSRs in the cp genome present high diversity in copy numbers, and are important molecular markers for plant population genetics, and evolutionary, and ecological studies (Huang et al., 2014; Zhao et al., 2015). With MISA analysis, 116 SSRs with a length of at least 10 bp were detected in the five *Epimedium* cp genomes with 103 loci showing polymorphism (Table 3, Table S6, S7). Each *Epimedium* cp genome was found to contain 80 to 87 SSRs, of which 13 SSRs appeared same for the five cp genomes, and the numbers of polymorphic SSRs ranged from 67 to 74. Among the 116 SSRs, the mono-, di-, trin-, tetra-, penta-, and hexanucleotide SSRs were all detected, the mononucleotide SSRs were richest with a portion of 72.76%, and the mononucleotide A and T repeat units occupied the highest portion with 35.34% and 44.83%, respectively. These 116 SSR loci mainly located in intergenic spacer (IGS, 62.07%), following by pCDS (13.79%) and introns (23.28%). Only one SSR crossed the pCDS and IGS (*psbI-psbI/trnS-GCU*) in the cp genome of *E. acuminatum*. We observed that 16 SSRs located in 10 protein-coding genes [*rpoc2, rpoB, psbC, psaA, psbF, ycf2* (×4), *ycf1* (×4), *rpl32, ndhE, ndhH*] of the five *Epimeidum* cp genomes. Most of those SSR loci were located in LSC region, followed by SSC and IR regions. In general, the cp SSRs of the five *Epimedium* represented abundant variation, and undoubtedly useful for assays detecting polymorphisms at population-level as well as comparing more distantly phylogenetic relationships among *Epimedium* species.

**Table 3:** **Simple sequence repeats (SSRs) in the five ***Epimedium*** cp genomes**.

**Species**	**SSR loci no.**	**PolyM. loci no.**	**PolyM. loci (%).**	**P1 loci no.**	**P2 loci no.**	**P3 loci no.**	**P4 loci no.**	**P5 loci no.**	**P6 loci no.**	**Location**				**Region**		
										**IGS**	**Intron**	**pCDS**	**pCDS-IGS**	**LSC**	**SSC**	**IR**
*E. acuminatum*	87	74	85.06	73	6	/	5	1	2	54	19	13	1	73	10	4
*E. dolichostemon*	80	67	83.75	68	6	/	5	1	/	50	18	12	/	67	9	4
*E. lishihchenii*	87	74	85.06	74	6	1	6	/	/	51	22	14	/	71	12	4
*E. pseudowushanense*	84	71	84.52	71	6	/	6	1	/	50	21	13	/	71	9	4
*E. koreanum*	85	72	84.71	72	7	/	5	/	1	49	23	13	/	70	11	4
Total Loci	116	103	88.79	96	7	1	8	1	3	72	27	16	1	97	13	6

*P1 to P6 represented SSR loci with mono-, di-, tri-, tetra-, penta-, and hexanucleotide repeats, respectively*.

### Repetitive sequences

**Figure 5:**
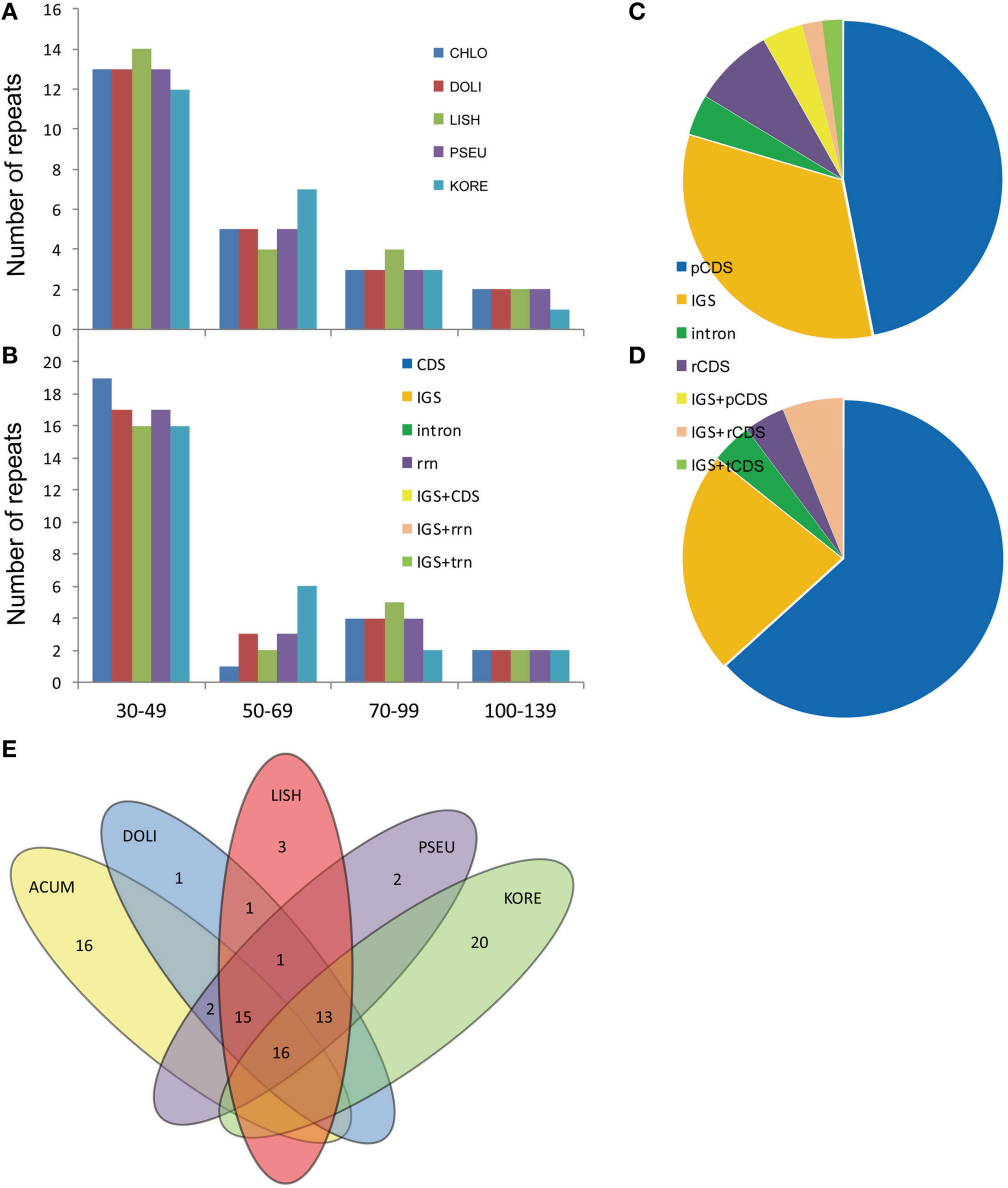
**Analysis of repeated sequences in the five ***Epimedium*** chloroplast genomes. (A)** Frequency of the direct repeats by length; **(B)**. Frequency of the palindromic repeats; **(C)**. Location of repeats in the four *Epimedium* cp genomes endemic to China; **(D)**. Location of repeats in the cp genome of *E. koreanum*; **(E)**. Summary of shared repeats among the five *Epimedium* chloroplast genomes. chlo, *E. acuminatum*; dewu, *E. dolichostemon*; lish, *E. lishihchenii*; pseu, *E. pseudowushanense*; kore, *E. koreanum*; IGS, intergenic spacer.

With the criterion of copy size 30 bp or longer and sequence identity >90%, REPuter identified a total of 49 repeats in the five *Epimedium* cp genomes, including direct, and palindromic repeats (Figure 5, Table S8). Except for *E. koreanum* with 24 direct repeats and 25 palindromic repeats, the other four *Epimedium* species identically possessed 23 direct repeats, and 26 palindromic repeats. The lengths of repeats in the five *Epimedium* cp genomes ranged from 31 to 131 bp, and the copy lengths with 30–49 bp are most common (61.22%) while those with more than 100 bp were least (7.76%). Under the criterion with identical lengths located in homologous regions as shared repeats, we investigated the repeats shared among the five *Epimedium* cp genomes. There were 16 repeats shared by the five *Epimedium* cp genomes, 15 repeats shared by the four *Epimedium* species endemic to China, 13 repeats shared by *E. koreanum* and the three of four Chinese *Epimedium* species, and four repeats shared by two or three Chinese *Epimedium* species. *E. koreanum* had the most unique repeats (20), followed with *E. acuminatum* (16), while the other three *Epimedium* species had one to three unique repeats. The repeats of the five *Epimedium* cp genomes were mainly located in pCDS and IGS, while the minority was located in intron and *rrn* gene coding region (rCDS), or covered across IGS and one of pCDS, rCDS, or *trn* gene coding region (tCDS). Except for *E. koreanum*, the proportions of repeat locations were identical in the other four *Epimedium* species.

Contrasting to the major repeats of most angiosperm plant cp genomes located in noncoding regions (Uthaipaisanwong et al., 2012; Yao et al., 2015), the proportions of repeats located in coding regions (CDS) were higher than those in noncoding regions in *Epimedium* species. In *E. koreanum* cp genomes, the proportion of the repeats located in pCDS led to 63.27%, while the repeats located in IGS only accounted for 22.45%. Previous work suggested that repeat sequences have played an important role in sequence rearranging and variation in cp genomes through illegitimate recombination and slipped-strand mispairing (Bausher et al., 2006; Saski et al., 2007; Huang et al., 2014). Our research also showed that divergent regions of cp genomes were associated with various repeat sequences such as *ycf1* gene and intergenic *trnQ-UUG*/*psbK*. These repeats may further serve as genetic markers for phylogenetic and population genetic studies on *Epimedium* species.

### Phylogenetic analysis

**Figure 6:**
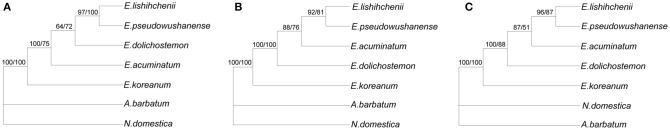
**Phylogenetic relationships of the five ***Epimedium*** species constructed by CDS regions (A), noncoding regions (B), and whole cp genome sequences (C) with maximum parsimony (MP) and maximum likelihood**. Numbers above node are bootstrap support values (>50%) with MP bootstrap values on the left and ML bootstrap on the right.

The cp genome sequences are addressed successfully for the phylogenetic studies of angiosperm (Jansen et al., 2007; Huang et al., 2014; Kim et al., 2015). In the present studies, three datasets (protein coding exons, introns and spacers, and whole complete cp genome sequences) from cp genomes of five *Epimedium* species and two outgroups were used to perform phylogenetic analysis. Among the three datasets, introns and spacers contained the highest parsimony informative characters (6.85%), followed by whole complete cp genome sequences (5.22%) and protein coding exons (5.03%). Using MP and ML analyses, phylogenetic trees were built based on the three datasets (Figure 6). The topologies based on both analyses were highly concordant in each dataset, as well as the dendrograms based on the noncoding sequences and whole complete cp genome sequences, and the phylogenetic trees of the three datasets were largely congruent with each other. For the five *Epimedium* species, *E. koreanum* is distributed in Northeast China, Japan, and Korea, and belongs to sect. *Macroceras*, while the other four species are native to Central and Southwest China, being attributed to sect. *Diphyllon* (Stearn, 2002). The resulting six phylogenetic trees identically exhibited that *E. koreanum* were firstly separated from the other four *Epimedium* species. For the four *Epimedium* species of sect. *Diphyllon, E. dolichostemon* has relatively small flowers and short spurs, being a member of ser. *Brachycerae*; the other three species has large flowers with petals bearing long spurs, of which *E. acuminatum* and *E. lishihchenii* possess petal without basal laminae, being attributed to ser. *Dolichocerae*, while *E. pseudowushanense* possesses petal with slight basal lamina, belonging to ser. *Davidianae*. In accordance with classical taxonomy of *Epimedium* (Stearn, 2002), phylogenetic trees based on noncoding regions and whole complete cp genome sequences all supported that *E. dolichostemon* was early divided from the other three species of sect. *Diphyllon*. However, the basal position of *E. dolichostemon* among four species of sec. *Diphyllon* was inconsistent with Stearn's (2002) and Ying's (2002) interpretation on floral evolution of the genus. Furthermore, all trees based on the three datasets identically supported that *E. lishihchenii* firstly clustered with *E. pseudowushanense*, not with *E. acuminatum* from the same series, which were coincident with the previous phylogenetic studies based on AFLPs (Zhang et al., 2014). These results showed that Stearn's (2002) taxonomic system of *Epimedium* is reasonable on the whole and the phylogenetic relationships within Chinese sect. *Diphyllon* are closely related with corolla characters, especially with petals. However, the evolutionary relationships and the divisions within the section need further investigation applying more evidences.

## Author contributions

YZ, YW, and TY conceived and designed the experiment, and writed the paper. JC, WZ, AL, KK, and SL collected the materials. KK, SL, YZ, and LD performed the experiments. KK and SL completed the sequence assembly. LD, YZ, LW, and WH conducted the comprehensive analyses on the cp genome sequences.

### Conflict of interest statement

The authors declare that the research was conducted in the absence of any commercial or financial relationships that could be construed as a potential conflict of interest.
