# Supplementary material for: The Complete Chloroplast Genome Sequences of Five Epimedium Species: Lights into Phylogenetic and Taxonomic Analyses
Source: Front Plant Sci. 2016 Mar 15;7:306. doi: 10.3389/fpls.2016.00306 (PMC4791396; doi:10.3389/fpls.2016.00306)
Supplement: Supplementary file 1 [file Table1.DOCX]

Table S1. List of taxa included in this study.

| Species | Origin | Voucher / Reference |
| --- | --- | --- |
| *E. acuminatum* | China, Sichuan, Baoxing | *Y.J. Zhang 313* (HIB) |
| *E. dolichostemon* | China, Guizhou, Dejiang | *Y.J. Zhang 163* (HIB) |
| *E. lishihchenii* | China, Jiangxi, Lushan | *Y.J. Zhang 233* (HIB) |
| *E. pseudowushanense* | China, Guizhou, Leishan | *Y.J. Zhang 425* (HIB) |
| *E. koreanum* | South Korea, Gangwon-do, Mt. Yong-hwa | Lee et al. (2015). |
