# Supplementary material for: The Complete Chloroplast Genome Sequences of Five Epimedium Species: Lights into Phylogenetic and Taxonomic Analyses
Source: Front Plant Sci. 2016 Mar 15;7:306. doi: 10.3389/fpls.2016.00306 (PMC4791396; doi:10.3389/fpls.2016.00306)
Supplement: Supplementary file 2 [file Table2.DOCX]

Table S2 Primers used for genome sequence validation.

| **Primer** | **location** | **Forward sequence** | **Reverse sequence** |
| --- | --- | --- | --- |
| P01 | LSC/IRA | CACCGGCGGATATTCTCATATT | ACTAGACCTTTGGTCGTCAATAC |
| P02 | IRA/SSC | GATAAGACAGATAAGACTGCCGAG | CCCAGGAATCGGACAATACAA |
| P03 | IRB/SSC | ATTTAGTACAGGAGCCTTCGC | CGATTTGGACTCAAGCATCAAC |
| P04 | IRB/LSC | GTTAAAGCGCCCAACTCATAATTG | ATTCGTATTAGTGTATTGTTTTCGAATG |
| P05 | *infA* | TTCCAACGAACGGGATCTTC | GCCTACGAATCTATTCCCACTATC |
| P06 | *ndhF* | ACGACAACGAAGACAGAGATTAC | GTGGCATAAGAAGGTTAGCAGA |
| P07 | *psbC* | CCAGAGAAGCCCATGTATGAA | TGCCAGATTCCGCCAAATA |
| P08 | *psbK* | CCCGTTTATCCCTTAATCAATAACTC | AGCAGCTTGCCAAACAAAG |
| P09 | *rpoA* | CTCCGTACAGTGCCCAATAA | ATGCGGAAGAACAAGACATAGA |
| P10 | *rps15* | GCGACTCAGTTCCAGCATAA | ATCGGCAGGCTCTTCTTAATC |
| P11 | *rps16* | TGCTGCTGCACCACATAA | GAGATGGAAGGGCTCTTTGTAA |
| P12 | *rps16* | GACGAGTGAATAGAGTGACTTTGA | AGGTAAGGTAAGAGGGTTGGA |
| P13 | *psaA* | GGATGCCTGTGCCCATAAA | AGGGACTACGATCCAACTACTC |
| P14 | *rpl32* | AACACGACGAAACTATCTGGAG | AGAATCCTGAGTAGCCATTTACC |
