# Supplementary material for: The Complete Chloroplast Genome Sequences of Five Epimedium Species: Lights into Phylogenetic and Taxonomic Analyses
Source: Front Plant Sci. 2016 Mar 15;7:306. doi: 10.3389/fpls.2016.00306 (PMC4791396; doi:10.3389/fpls.2016.00306)
Supplement: Supplementary file 3 [file Table3.DOCX]

Table S3. Accession numbers of chloroplast genome sequences included in phylogenetic analyses.

| **Order** | **Family** | **Taxon** | **Accession No.** |
| --- | --- | --- | --- |
| Ranales | Ranunculales | *Ranunculus macranthus* | DQ359689 |
| Ranales | Ranunculales | *Aconitum barbatum* | KC844054 |
| Ranales | Ranunculales | *Megaleranthis saniculifolia* | FJ597983 |
| Ranales | Berberidaceae | *Nandina domestica* | DQ923117 |
| Ranales | Berberidaceae | *Mahonia bealei* | KF176554 |
| Proteales | Platanaceae | *Platanus occidentalis* | NC_008335 |
| Proteales | Nelumbonaceae | *Nelumbo nucifera* | JQ336993 |
| Proteales | Nelumbonaceae | *Nelumbo lutea* | NC_015605 |
| Buxales | Buxaceae | *Buxus microphylla* | NC_009599 |
| Trochodendrales | Tetracentraceae | *Tetracentron sinense* | KC608752 |
| Trochodendrales | Trochodendraceae | *Trochodendron aralioides* | KC608753 |
