# Supplementary material for: The Complete Chloroplast Genome Sequences of Five Epimedium Species: Lights into Phylogenetic and Taxonomic Analyses
Source: Front Plant Sci. 2016 Mar 15;7:306. doi: 10.3389/fpls.2016.00306 (PMC4791396; doi:10.3389/fpls.2016.00306)
Supplement: Supplementary file 4 [file Table4.docx]

Table S4. The sequence divergence analysis on the protein-coding regions of the five *Epimedium* chloroplast genomes.

| Protein-coding region | Characters(n) | Polymorphic characters (n) | Divergence proportion | Location |
| --- | --- | --- | --- | --- |
| *rps16* | 445 | 143 | 0.321348 | LSC |
| *psbK* | 186 | 44 | 0.236559 | LSC |
| *rpl32* | 163 | 32 | 0.196319 | IRa, b |
| *rps14* | 334 | 31 | 0.092814 | LSC |
| *rps15* | 282 | 10 | 0.035461 | SSC |
| *ycf1* | 5793 | 150 | 0.025893 | SSC/IRa,b |
| *rpoA* | 1012 | 18 | 0.017787 | LSC |
| *accD* | 1533 | 27 | 0.017613 | LSC |
| *ndhF* | 2244 | 34 | 0.015152 | SSC |
| *rps18* | 334 | 5 | 0.01497 | LSC |
| *rpl14* | 369 | 5 | 0.01355 | LSC |
| *ndhE* | 306 | 4 | 0.013072 | SSC |
| *ycf2* | 7137 | 75 | 0.010509 | IRa, b |
| *rps8* | 399 | 4 | 0.010025 | LSC |
| *clpP* | 627 | 6 | 0.009569 | LSC |
| *psaA* | 2253 | 21 | 0.009321 | LSC |
| *psbH* | 228 | 2 | 0.008772 | LSC |
| *rpl36* | 114 | 1 | 0.008772 | LSC |
| *psbL* | 117 | 1 | 0.008547 | LSC |
| *rpl20* | 354 | 3 | 0.008475 | LSC |
| *ndhA* | 1095 | 8 | 0.007306 | SSC |
| *cemA* | 690 | 5 | 0.007246 | LSC |
| *rps2* | 711 | 5 | 0.007032 | LSC |
| *matK* | 1533 | 10 | 0.006523 | LSC |
| *rpl23* | 309 | 2 | 0.006472 | IRa, b, or LSC/IRa, b |
| *petA* | 969 | 6 | 0.006192 | LSC |
| *atpF* | 555 | 3 | 0.005405 | LSC |
| *psbZ* | 189 | 1 | 0.005291 | LSC |
| *atpA* | 1524 | 8 | 0.005249 | LSC |
| *rpl2* | 825 | 4 | 0.004848 | IRa, b, or LSC/IRa, b |
| *ndhD* | 1503 | 7 | 0.004657 | SSC |
| *rps3* | 666 | 3 | 0.004505 | LSC |
| *ndhH* | 1182 | 5 | 0.00423 | SSC |
| *rbcL* | 1428 | 6 | 0.004202 | LSC |
| *ndhJ* | 477 | 2 | 0.004193 | LSC |
| *ccsA* | 966 | 4 | 0.004141 | SSC |
| *rpoC2* | 4137 | 17 | 0.004109 | LSC |
| *atpH* | 246 | 1 | 0.004065 | LSC |
| *psbE* | 252 | 1 | 0.003968 | LSC |
| *rpoC1* | 2034 | 8 | 0.003933 | LSC |
| *petB* | 648 | 2 | 0.003086 | LSC |
| *rps7* | 324 | 1 | 0.003086 | IRa, b |
| *ndhK* | 672 | 2 | 0.002976 | LSC |
| *rpoB* | 3216 | 9 | 0.002799 | LSC |
| *rps12* | 372 | 1 | 0.002688 | LSC, IRa, b |
| *atpE* | 402 | 1 | 0.002488 | LSC |
| *rps11* | 423 | 1 | 0.002364 | LSC |
| *psaB* | 2205 | 5 | 0.002268 | LSC |
| *petD* | 504 | 1 | 0.001984 | LSC |
| *rpl22* | 519 | 1 | 0.001927 | LSC/IRa, b |
| *ndhG* | 531 | 1 | 0.001883 | SSC |
| *psbD* | 1062 | 2 | 0.001883 | LSC |
| *rps4* | 624 | 1 | 0.001603 | LSC |
| *atpI* | 744 | 1 | 0.001344 | LSC |
| *atpB* | 1497 | 2 | 0.001336 | LSC |
| *psbB* | 1527 | 2 | 0.00131 | LSC |
| *psbA* | 1062 | 1 | 0.000942 | LSC |
| *psbC* | 1422 | 1 | 0.000703 | LSC |
| *ndhB* | 1536 | 1 | 0.000651 | IRa, b |
| *ndhC* | 363 | 0 | 0 | LSC |
| *ndhI* | 537 | 0 | 0 | SSC |
| *petG* | 114 | 0 | 0 | LSC |
| *petL* | 96 | 0 | 0 | LSC |
| *petN* | 90 | 0 | 0 | LSC |
| *psaC* | 246 | 0 | 0 | SSC |
| *psaI* | 111 | 0 | 0 | LSC |
| *psaJ* | 135 | 0 | 0 | LSC |
| *psbF* | 120 | 0 | 0 | LSC |
| *psbI* | 111 | 0 | 0 | LSC |
| *psbJ* | 123 | 0 | 0 | LSC |
| *psbM* | 105 | 0 | 0 | LSC |
| *psbN* | 132 | 0 | 0 | LSC |
| *psbT* | 108 | 0 | 0 | LSC |
| *rpl16* | 441 | 0 | 0 | LSC |
| *rpl33* | 201 | 0 | 0 | LSC |
| *rps19* | 279 | 0 | 0 | IRa, b, or LSC |
| *ycf3* | 507 | 0 | 0 | LSC |
| *ycf4* | 555 | 0 | 0 | LSC |
