# Supplementary material for: The Complete Chloroplast Genome Sequences of Five Epimedium Species: Lights into Phylogenetic and Taxonomic Analyses
Source: Front Plant Sci. 2016 Mar 15;7:306. doi: 10.3389/fpls.2016.00306 (PMC4791396; doi:10.3389/fpls.2016.00306)
Supplement: Supplementary file 5 [file Table5.docx]

Table S5. The sequence divergence analysis on the noncoding regions of the five *Epimedium* chloroplast genomes, including introns and spacers.

| Intron and spacer | Character | Polymorphic character | Divergence proportion | Location |
| --- | --- | --- | --- | --- |
| *rpl23*/*rpl2*_extron_1 | 1 | 1 | 1 | IRb |
| *rpl2*_extron_1/*rpl2*_extron_2 | 672 | 672 | 1 | IRb |
| *rpl2*_extron_2/*rps19* | 98 | 98 | 1 | IRb |
| *rps19*/ψ*rpl22* | 53 | 53 | 1 | IRb |
| *ycf1*/*ndhF* | 292 | 253 | 0.865517 | SSC |
| *trnC-GCA*/*petN* | 736 | 385 | 0.523098 | LSC |
| ψ*rpl22*, ψ*rpl2*, ψ*rpl23*/*trnH-GUG* | 93 | 45 | 0.483871 | LSC |
| *trnQ-UUG*/*psbK* | 465 | 119 | 0.255914 | LSC |
| *rpl36/rps8* | 444 | 100 | 0.225225 | LSC |
| *rps12*_5'end/*clpP*_extron_3 | 1256 | 261 | 0.207803 | LSC |
| *rps19*/*rpl2*_extron_2 | 98 | 18 | 0.183673 | IRa, or LSC |
| *rps8*/*rpl14* | 222 | 37 | 0.166667 | LSC |
| *trnG-UCC*_extron_2/*trnR-UCU* | 194 | 27 | 0.139175 | LSC |
| *trnK-UUU*_extron_1/*rps16* | 1478 | 201 | 0.135995 | LSC |
| *psbE*/*petL* | 720 | 90 | 0.125 | LSC |
| *rpl32*/*trnL-UAG* | 824 | 103 | 0.125 | SSC |
| *rps15*/*ycf1* | 456 | 52 | 0.114035 | SSC |
| *ndhH*/*rps15* | 53 | 6 | 0.113208 | SSC |
| *psbK*/*psbI* | 438 | 48 | 0.109589 | LSC |
| *ndhF*/*rpl32* | 707 | 77 | 0.108911 | SSC |
| *petD*/*rpoA* | 217 | 22 | 0.101382 | LSC |
| *psbI*/*trnS-GCU* | 167 | 16 | 0.095808 | LSC |
| *trnL-UAG*/*ccsA* | 84 | 8 | 0.095238 | SSC |
| *ccsA*/*ndhD* | 212 | 20 | 0.09434 | SSC |
| *psbT*/*psbN* | 75 | 7 | 0.093333 | LSC |
| *psaI*/*ycf4* | 197 | 18 | 0.091371 | LSC |
| *trnD-GUC*/*trnY-GUA* | 309 | 28 | 0.090615 | LSC |
| *ndhD*/*psaC* | 136 | 12 | 0.088235 | SSC |
| *accD*/*psaI* | 871 | 74 | 0.08496 | LSC |
| *trnI-CAU*/*ycf2* | 175 | 13 | 0.074286 | IRa, b |
| *ndhJ*/*ndhK* | 116 | 8 | 0.068966 | LSC |
| *psbC*/*trnS-UGA* | 231 | 14 | 0.060606 | LSC |
| *rps14*/*psaB* | 135 | 8 | 0.059259 | LSC |
| *trnH-GUG*/*psbA* | 442 | 25 | 0.056561 | LSC |
| *petA*/*psbJ* | 637 | 36 | 0.056515 | LSC |
| *trnS-GCU*/*trnG-UCC* | 459 | 25 | 0.054466 | LSC |
| *petN*/*psbM* | 1129 | 59 | 0.052259 | LSC |
| *atpI*/*rps2* | 212 | 11 | 0.051887 | LSC |
| *ycf4*/*cemA* | 167 | 8 | 0.047904 | LSC |
| *psaJ*/*rpl33* | 446 | 21 | 0.047085 | LSC |
| *rps18*/*rpl20* | 258 | 12 | 0.046512 | LSC |
| *clpP*_extron_1/*psbB* | 432 | 20 | 0.046296 | LSC |
| *trnT-UGU*/*trnL-UAA*_extron_1 | 871 | 40 | 0.045924 | LSC |
| *trnS-UGA*/*psbZ* | 357 | 16 | 0.044818 | LSC |
| *psbZ*/*trnG-GCC* | 448 | 20 | 0.044643 | LSC |
| *ndhA*_extron_2/*ndhA*_extron_1 | 1020 | 45 | 0.044118 | SSC |
| *rpl16*_extron_2/*rpl16*_extron_1 | 1183 | 49 | 0.04142 | LSC |
| *atpH*/*atpI* | 1132 | 45 | 0.039753 | LSC |
| *trnF-GAA*/*ndhJ* | 865 | 32 | 0.036994 | LSC |
| *matK*/*trnK-UUU*_extron_1 | 760 | 28 | 0.036842 | LSC |
| *ndhK*/*ndhC* | 56 | 2 | 0.035714 | LSC |
| *trnY-GUA*/*trnE-UUC* | 59 | 2 | 0.033898 | LSC |
| *rbcL*/*accD* | 716 | 24 | 0.03352 | LSC |
| *ndhE*/*ndhG* | 209 | 7 | 0.033493 | SSC |
| *ndhC*/*trnV-UAC*_extron_2 | 1698 | 54 | 0.031802 | LSC |
| *rpl16*_extron_1/*rps3* | 198 | 6 | 0.030303 | LSC |
| *atpF*_extron_1/*atpH* | 504 | 15 | 0.029762 | LSC |
| *rps16*_extron_2/*rps16*_extron_1 | 811 | 22 | 0.027127 | LSC |
| *atpB*/*rbcL* | 726 | 18 | 0.024793 | LSC |
| *psaA*/*ycf3*_extron_3 | 772 | 19 | 0.024611 | LSC |
| *petL*/*petG* | 163 | 4 | 0.02454 | LSC |
| *trnG-GCC*/*trnfM-CAU* | 164 | 4 | 0.02439 | LSC |
| *rpoB*/*trnC-GCA* | 1161 | 28 | 0.024117 | LSC |
| *clpP*_extron_3/*clpP*_extron_2 | 665 | 16 | 0.02406 | LSC |
| *rpl14*/*rpl16*_extron_2 | 85 | 2 | 0.023529 | LSC |
| *trnL-UAA*_extron_2/*trnF-GAA* | 356 | 8 | 0.022472 | LSC |
| *trnP-UGG*/*psaJ* | 358 | 8 | 0.022346 | LSC |
| *trnT-GGU*/*psbD* | 1168 | 26 | 0.02226 | LSC |
| *ycf3*_extron_2/*ycf3*_extron_1 | 740 | 16 | 0.021622 | LSC |
| *ndhG*/*ndhI* | 375 | 7 | 0.018667 | SSC |
| *petB*_extron_2/*petD*_extron_1 | 219 | 4 | 0.018265 | LSC |
| *rpl33*/*rps18* | 171 | 3 | 0.017544 | LSC |
| *psbJ*/*psbL* | 116 | 2 | 0.017241 | LSC |
| *clpP*_extron_2/*clpP*_extron_1 | 932 | 16 | 0.017167 | LSC |
| *ndhB*_extron_1/*rps7* | 426 | 7 | 0.016432 | IRa, b |
| *psbH*/*petB*_extron_1 | 123 | 2 | 0.01626 | LSC |
| *rpoA*/*rps11* | 65 | 1 | 0.015385 | LSC |
| *trnV-UAC*_extron_2/*trnV-UAC*_extron_1 | 589 | 9 | 0.01528 | LSC |
| *atpA*/*atpF* | 66 | 1 | 0.015152 | LSC |
| *trnE-UUC*/*trnT-GGU* | 681 | 10 | 0.014684 | LSC |
| *psbA*/*trnK-UUU* | 221 | 3 | 0.013575 | LSC |
| *rps4*/*trnT-UGU* | 369 | 5 | 0.01355 | LSC |
| *rpoC2*/*rpoC1*_extron_2 | 175 | 2 | 0.011429 | LSC |
| *trnS-GGA*/*rps4* | 277 | 3 | 0.01083 | LSC |
| *trnQ-UUG*/*trnQ-UUG* | 101 | 1 | 0.009901 | LSC |
| *psbN*/*psbH* | 102 | 1 | 0.009804 | LSC |
| *atpF*_extron_2/*atpF*_extron_1 | 718 | 7 | 0.009749 | LSC |
| *petB*_extron_1/*petB*_extron_2 | 824 | 8 | 0.009709 | LSC |
| *ndhI*/*ndhA*_extron_2 | 103 | 1 | 0.009709 | SSC |
| *psbM*/*trnD-GUC* | 1156 | 11 | 0.009516 | LSC |
| *trnL-UAA*_extron_1/*trnL-UAA*_extron_2 | 425 | 4 | 0.009412 | LSC |
| *ycf3*_extron_1/*trnS-GGA* | 862 | 8 | 0.009281 | LSC |
| *ycf3*_extron_3/*ycf3*_extron_2 | 766 | 7 | 0.009138 | LSC |
| *cemA*/*petA* | 222 | 2 | 0.009009 | LSC |
| *rpl2*_extron_2/*rpl2*_extron_1 | 672 | 6 | 0.008929 | IRa, or LSC |
| *trnM-CAU*/*atpE* | 230 | 2 | 0.008696 | LSC |
| *trnG-UCC*_extron_1/ *trnG-UCC*_extron_2 | 698 | 6 | 0.008596 | LSC |
| *rpl20*/*rps12*_5'end | 789 | 6 | 0.007605 | LSC |
| *trnL-CAA*/*ndhB*_extron_2 | 551 | 4 | 0.00726 | IRa, b |
| *petD*_extron_1/*petD*_extron_2 | 749 | 5 | 0.006676 | LSC |
| *trnfM-CAU*/*rps14* | 151 | 1 | 0.006623 | LSC |
| *trnW-CCA*/*trnP-UGG* | 160 | 1 | 0.00625 | LSC |
| *rps2*/*rpoC2* | 213 | 1 | 0.004695 | LSC |
| *rrn4.5*/*rrn5* | 225 | 1 | 0.004444 | IRa, b |
| *psaC*/*ndhE* | 240 | 1 | 0.004167 | SSC |
| *trnK-UUU*_extron_2/*matK* | 256 | 1 | 0.003906 | LSC |
| *rrn5*/*trnR-ACG* | 266 | 1 | 0.003759 | IRa, b |
| *trnI-GAU*_extron_1/*trnI-GAU*_extron_2 | 953 | 3 | 0.003148 | IRa, b |
| *rpoC1*_extron_2/*rpoC1*_extron_1 | 738 | 2 | 0.00271 | LSC |
| *trnR-ACG*/*trnN-GUU* | 600 | 1 | 0.001667 | IRa, b |
| *ycf2*/*trnL-CAA* | 931 | 1 | 0.001074 | IRa, b |
| *rps16*_extron_1/*trnQ-UUG* | 140 | 0 | 0 | LSC |
| *trnR-UCU*/*atpA* | 143 | 0 | 0 | LSC |
| *rpoC1*_extron_1/*rpoB* | 26 | 0 | 0 | LSC |
| *psbD*/*psbC* | 55 | 0 | 0 | LSC |
| *psaB*/*psaA* | *25* | *0* | *0* | LSC |
| *trnV-UAC*_extron_1/*trnM-CAU* | 185 | 0 | 0 | LSC |
| *atpE*/*atpB* | 6 | 0 | 0 | LSC |
| *psbL*/*psbF* | 27 | 0 | 0 | LSC |
| *psbF*/*psbE* | 9 | 0 | 0 | LSC |
| *petG*/*trnW-CCA* | 131 | 0 | 0 | LSC |
| *psbB*/*psbT* | 183 | 0 | 0 | LSC |
| *rps11*/*rpl36* | 113 | 0 | 0 | LSC |
| *rps3*/*rpl22* | 18 | 0 | 0 | LSC |
| *rpl22*/*rps19* | 53 | 0 | 0 | IRa, or LSC |
| *rpl2*_extron_1/*rpl23* | 1 | 0 | 0 | IRa, or LSC |
| *rpl23*/*trnI-CAU* | 173 | 0 | 0 | IRa, b |
| *ndhB*_extron_2/*ndhB*_extron_1 | 700 | 0 | 0 | IRa, b |
| *rps7*/*rps12*_3'end_extron_3 | 58 | 0 | 0 | IRa, b |
| *rps12*_3'end_extron_3/*rps12*_3'end_extron_2 | 536 | 0 | 0 | IRa, b |
| *rps12*_3'end_extron_2/*trnV-GAC* | 1333 | 0 | 0 | IRa, b |
| *trnV-GAC*/*rrn16* | 231 | 0 | 0 | IRa, b |
| *rrn16*/*trnI-GAU*_extron_1 | 298 | 0 | 0 | IRa, b |
| *trnI-GAU*_extron_2/*trnA-UGC*_extron_1 | 64 | 0 | 0 | IRa, b |
| *trnA-UGC*_extron_1/*trnA-UGC*_extron_2 | 800 | 0 | 0 | IRa, b |
| *trnA-UGC*_extron_2/*rrn23* | 158 | 0 | 0 | IRa, b |
| *rrn23*/*rrn4.5* | 98 | 0 | 0 | IRa, b |
| *trnN-GUU*/*ycf1* | 147 | 0 | 0 | IRa, b |
| *ndhA*_extron_1/*ndhH* | 1 | 0 | 0 | SSC |
