# Supplementary material for: The Complete Chloroplast Genome Sequences of Five Epimedium Species: Lights into Phylogenetic and Taxonomic Analyses
Source: Front Plant Sci. 2016 Mar 15;7:306. doi: 10.3389/fpls.2016.00306 (PMC4791396; doi:10.3389/fpls.2016.00306)
Supplement: Supplementary file 6 [file Table6.DOCX]

Table S6. Distribution of simple sequence repeats (SSRs) loci in the five *Epimedium* chloroplast genomes. SSR locus with * in the column of SSR No. indicated that the SSR was same in nucleotide unit and the number of the repeat unit for the each of five *Epimedium* cp genomes.

| Genomes/SSR No. | repeat unit | No. repeat unit | SSR size | Position A | Position B | Region | Location | Locus |
| --- | --- | --- | --- | --- | --- | --- | --- | --- |
| *E. acuminatum* |  |  |  |  |  |  |  |  |
| 1 | A | 10 | 10 | 264 | 273 | *trnH-GUG/psbA* | IGS | LSC |
| 2 | A | 10 | 10 | 4025 | 4034 | *matK/trnK-UUU* | IGS | LSC |
| 3 | A | 10 | 10 | 13699 | 13708 | *atpF/atpH* | IGS | LSC |
| 4* | A | 10 | 10 | 15083 | 15092 | *atpH/atpI* | IGS | LSC |
| 5 | A | 10 | 10 | 28213 | 28222 | *rpoB/trnC-GCA* | IGS | LSC |
| 6 | A | 10 | 10 | 28337 | 28346 | *rpoB/trnC-GCA* | IGS | LSC |
| 7 | A | 10 | 10 | 45924 | 45933 | *ycf3/trnS-GGA* | IGS | LSC |
| 8 | A | 10 | 10 | 66306 | 66315 | *psbF* | pCDS | LSC |
| 9 | A | 10 | 10 | 68842 | 68851 | *psaJ/rpl33* | IGS | LSC |
| 10 | A | 10 | 10 | 65338 | 65347 | *petA/psbJ* | IGS | LSC |
| 11 | A | 10 | 10 | 78090 | 78099 | *petB* | Intron | LSC |
| 12 | A | 11 | 11 | 4868 | 4878 | *trnK-UUU/rps16* | IGS | LSC |
| 13 | A | 11 | 11 | 8608 | 8618 | *psbI/trnS-GCU* | IGS | LSC |
| 14 | A | 11 | 11 | 13040 | 13050 | *atpF* | Intron | LSC |
| 15 | A | 11 | 11 | 29151 | 29161 | *petN/psbM* | IGS | LSC |
| 16 | A | 11 | 11 | 8518 | 8528 | *psbI, psbI/trnS-GCU* | pCDS-IGS | LSC |
| 17 | A | 11 | 11 | 72886 | 72896 | *clpP* | Intron | LSC |
| 18 | A | 11 | 11 | 82802 | 82812 | *rpl36/rps8* | IGS | LSC |
| 19 | A | 11 | 11 | 43773 | 43783 | *psaA/ycf3* | IGS | LSC |
| 20 | A | 11 | 11 | 71462 | 71472 | *rps12/clpP* | IGS | LSC |
| 21 | A | 12 | 12 | 8058 | 8069 | *psbK/psbI* | IGS | LSC |
| 22* | A | 12 | 12 | 57634 | 57645 | *atpB/rbcL* | IGS | LSC |
| 23 | A | 12 | 12 | 116911 | 116922 | *ndhF/rpl32* | IGS | SSC |
| 24 | A | 12 | 12 | 66935 | 66946 | *psbE/petL* | IGS | LSC |
| 25 | A | 12 | 12 | 8835 | 8846 | *trnS-GCU/trnG-UCC* | IGS | LSC |
| 26 | A | 13 | 13 | 38559 | 38571 | *rps14/psaB* | IGS | LSC |
| 27 | A | 13 | 13 | 68263 | 68275 | *trnP-UGG/psaJ* | IGS | LSC |
| 28 | A | 13 | 13 | 45666 | 45678 | *ycf3* | Intron | LSC |
| 29 | A | 13 | 13 | 124742 | 124754 | *ndhA* | Intron | SSC |
| 30 | A | 13 | 13 | 112798 | 112810 | *ycf1* | pCDS | IRB |
| 31 | A | 13 | 13 | 72339 | 72351 | *rps12/clpP* | IGS | LSC |
| 32 | A | 14 | 14 | 84422 | 84435 | *rpl16* | Intron | LSC |
| 33 | A | 15 | 15 | 61962 | 61976 | *accD/psaI* | IGS | LSC |
| 34 | A | 16 | 16 | 131282 | 131297 | *ycf1* | pCDS | SSC |
| 35 | T | 10 | 10 | 6554 | 6563 | *rps16* | Intron | LSC |
| 36 | T | 10 | 10 | 7486 | 7495 | *trnQ-UUG/psbK* | IGS | LSC |
| 37 | T | 10 | 10 | 30303 | 30312 | *psbM/trnD-GUC* | IGS | LSC |
| 38* | T | 10 | 10 | 5618 | 5627 | *trnK-UUU/rps16* | IGS | LSC |
| 39* | T | 10 | 10 | 18867 | 18876 | *rpoC2* | pCDS | LSC |
| 40 | T | 10 | 10 | 26655 | 26664 | *rpoB* | pCDS | LSC |
| 41* | T | 10 | 10 | 54202 | 54211 | *trnV-UAC* | Intron | LSC |
| 42 | T | 10 | 10 | 85301 | 85310 | *rpl16* | Intron | LSC |
| 43 | T | 10 | 10 | 127525 | 127534 | *rps15/ycf1* | IGS | SSC |
| 44 | T | 10 | 10 | 43173 | 43182 | *psaA/ycf3* | IGS | LSC |
| 45 | T | 10 | 10 | 73992 | 74001 | *clpP* | Intron | LSC |
| 46 | T | 10 | 10 | 77851 | 77860 | *petB* | Intron | LSC |
| 47 | T | 10 | 10 | 85578 | 85587 | *rpl16/rps3* | IGS | LSC |
| 48 | T | 10 | 10 | 73780 | 73789 | *clpP* | Intron | LSC |
| 49 | T | 10 | 10 | 82762 | 82771 | *rpl36/rps8* | pCDS | LSC |
| 50 | T | 11 | 11 | 23006 | 23016 | *rpoC1* | Intron | LSC |
| 51 | T | 11 | 11 | 29571 | 29581 | *petN/psbM* | IGS | LSC |
| 52 | T | 11 | 11 | 45597 | 45607 | *ycf3* | Intron | LSC |
| 53 | T | 11 | 11 | 78223 | 78233 | *petB* | Intron | LSC |
| 54 | T | 11 | 11 | 116786 | 116796 | *ndhF/rpl32* | IGS | SSC |
| 55 | T | 11 | 11 | 63283 | 63293 | *ycf4/cemA* | IGS | LSC |
| 56 | T | 11 | 11 | 117556 | 117566 | *rpl32* | pCDS | SSC |
| 57 | T | 11 | 11 | 50612 | 50622 | *trnF-GAA/ndhJ* | IGS | LSC |
| 58 | T | 11 | 11 | 47678 | 47688 | *rps4/trnT-UGU* | IGS | LSC |
| 59 | T | 11 | 11 | 65695 | 65705 | *petA/psbJ* | IGS | LSC |
| 60 | T | 12 | 12 | 8632 | 8643 | *psbI/trnS-GCU* | IGS | LSC |
| 61 | T | 12 | 12 | 31729 | 31740 | *trnD-GUC/trnY-GUA* | IGS | LSC |
| 62 | T | 12 | 12 | 72017 | 72028 | *rps12/clpP* | IGS | LSC |
| 63 | T | 12 | 12 | 85051 | 85062 | *rpl16* | Intron | LSC |
| 64 | T | 13 | 13 | 83313 | 83325 | *rps8/rpl14* | IGS | LSC |
| 65 | T | 13 | 13 | 132864 | 132876 | *ycf1* | pCDS | IRA |
| 66* | T | 13 | 13 | 117901 | 117913 | *rpl32/trnL-UAG* | IGS | SSC |
| 67 | T | 13 | 13 | 68986 | 68998 | *psaJ/rpl33* | IGS | LSC |
| 68 | T | 14 | 14 | 73047 | 73060 | *clpP* | Intron | LSC |
| 69 | T | 14 | 14 | 54068 | 54081 | *ndhC/trnV-UAC* | IGS | LSC |
| 70 | T | 16 | 16 | 67006 | 67021 | *psbE/petL* | IGS | LSC |
| 71* | C | 10 | 10 | 41529 | 41538 | *psaA* | pCDS | LSC |
| 72* | G | 10 | 10 | 57646 | 57655 | *atpB/rbcL* | IGS | LSC |
| 73 | G | 11 | 11 | 35527 | 35537 | *psbC* | pCDS | LSC |
| 74 | AT | 5 | 10 | 27439 | 27448 | *rpoB/trnC-GCA* | IGS | LSC |
| 75 | AT | 8 | 16 | 117776 | 117791 | *rpl32/trnL-UAG* | IGS | SSC |
| 76* | GA | 5 | 10 | 48938 | 48947 | *trnT-UGU/trnL-UAA* | IGS | LSC |
| 77 | TA | 6 | 12 | 49970 | 49981 | *trnF-GAA/ndhJ* | IGS | LSC |
| 78* | TA | 5 | 10 | 73944 | 73953 | *clpP* | Intron | LSC |
| 79* | TC | 5 | 10 | 126756 | 126765 | *ndhH* | pCDS | SSC |
| 80* | AGAA | 3 | 12 | 27471 | 27482 | *rpoB/trnC-GCA* | IGS | LSC |
| 81 | AGAA | 3 | 12 | 33797 | 33808 | *trnT-GGU/psbD* | IGS | LSC |
| 82* | ATTC | 3 | 12 | 77654 | 77665 | *petB* | Intron | LSC |
| 83 | TAAA | 3 | 16 | 52769 | 52784 | *ndhC/trnV-UAC* | IGS | LSC |
| 84 | TTGA | 3 | 12 | 121837 | 121848 | *ndhE* | pCDS | SSC |
| 85 | AGATA | 3 | 15 | 14794 | 14808 | *atpH/atpI* | IGS | LSC |
| 86 | ATCAAT | 3 | 18 | 150885 | 150902 | *ycf2* | pCDS | IRA |
| 87 | GATATT | 3 | 18 | 94769 | 94786 | *ycf2* | pCDS | IRB |
| *E. dolichostemon* |  |  |  |  |  |  |  |  |
| 1 | A | 10 | 10 | 8568 | 8577 | *psbI/trnS-GCU* | IGS | LSC |
| 2 | A | 10 | 10 | 13663 | 13672 | *atpF/atpH* | IGS | LSC |
| 3 | A | 10 | 10 | 29127 | 29135 | *petN/psbM* | IGS | LSC |
| 4 | A | 10 | 10 | 38512 | 38521 | *rps14/psaB* | IGS | LSC |
| 5 | A | 10 | 10 | 45859 | 45868 | *ycf3/trnS-GGA* | IGS | LSC |
| 6* | A | 10 | 10 | 66278 | 66287 | *psbF* | pCDS | LSC |
| 7 | A | 10 | 10 | 92004 | 92013 | *ycf2* | pCDS | IRB |
| 8 | A | 10 | 10 | 116788 | 116797 | *ndhF/rpl32* | IGS | SSC |
| 9 | A | 10 | 10 | 77984 | 77993 | *petB* | Intron | LSC |
| 10 | A | 11 | 11 | 4025 | 4035 | *matK/trnK-UUU* | IGS | LSC |
| 11 | A | 11 | 11 | 8024 | 8034 | *psbK/psbI* | IGS | LSC |
| 12 | A | 11 | 11 | 68853 | 68863 | *psaJ/rpl33* | IGS | LSC |
| 13 | A | 11 | 11 | 66907 | 66917 | *psbE/petL* | IGS | LSC |
| 14 | A | 11 | 11 | 53216 | 53226 | *ndhC/trnV-UAC* | IGS | LSC |
| 15 | A | 11 | 11 | 71483 | 71493 | *rps12/clpP* | IGS | LSC |
| 16 | A | 11 | 11 | 80556 | 80566 | *petD/rpoA* | IGS | LSC |
| 17 | A | 12 | 12 | 4861 | 4872 | *trnK-UUU/rps16* | IGS | LSC |
| 18 | A | 12 | 12 | 45602 | 45613 | *ycf3* | Intron | LSC |
| 19 | A | 13 | 13 | 15053 | 15065 | *atpH/atpI* | IGS | LSC |
| 20 | A | 13 | 13 | 84255 | 84267 | *rpl16* | Intron | LSC |
| 21 | A | 13 | 13 | 57575 | 57587 | *atpB/rbcL* | IGS | LSC |
| 22 | A | 13 | 13 | 124609 | 124621 | *ndhA* | Intron | SSC |
| 23* | A | 13 | 13 | 112680 | 112692 | *ycf1* | pCDS | IRB |
| 24 | A | 13 | 13 | 131166 | 131178 | *ycf1* | pCDS | SSC |
| 25 | A | 13 | 13 | 72234 | 72246 | *rps12/clpP* | IGS | LSC |
| 26 | A | 13 | 13 | 43708 | 43720 | *psaA/ycf3* | IGS | LSC |
| 27 | A | 14 | 14 | 72780 | 72793 | *clpP* | Intron | LSC |
| 28 | A | 14 | 14 | 61907 | 61920 | *accD/psaI* | IGS | LSC |
| 29 | A | 15 | 15 | 8792 | 8806 | *trnS-GCU/trnG-UCC* | IGS | LSC |
| 30 | T | 10 | 10 | 22979 | 22988 | *rpoC1* | Intron | LSC |
| 31 | T | 10 | 10 | 29545 | 29554 | *petN/psbM* | IGS | LSC |
| 32 | T | 10 | 10 | 36922 | 36931 | *trnS-UGA/psbZ* | IGS | LSC |
| 33 | T | 10 | 10 | 10050 | 10059 | *trnG-UCC/trnR-UCU* | IGS | LSC |
| 34* | T | 10 | 10 | 18840 | 18849 | *rpoC2* | pCDS | LSC |
| 35* | T | 10 | 10 | 26627 | 26636 | *rpoB* | pCDS | LSC |
| 36 | T | 10 | 10 | 45534 | 45543 | *ycf3* | Intron | LSC |
| 37* | T | 10 | 10 | 54143 | 54152 | trnV-UAC | Intron | LSC |
| 38 | T | 10 | 10 | 85138 | 85147 | *rpl16* | Intron | LSC |
| 39 | T | 10 | 10 | 127397 | 127406 | *rps15/ycf1* | IGS | SSC |
| 40 | T | 10 | 10 | 153421 | 153430 | *ycf2* | pCDS | IRA |
| 41 | T | 10 | 10 | 43108 | 43117 | *psaA/ycf3* | IGS | LSC |
| 42 | T | 10 | 10 | 63250 | 63259 | *ycf4/cemA* | IGS | LSC |
| 43 | T | 10 | 10 | 72038 | 72047 | *rps12/clpP* | IGS | LSC |
| 44 | T | 10 | 10 | 77745 | 77754 | *petB* | Intron | LSC |
| 45 | T | 10 | 10 | 50544 | 50553 | *trnF-GAA/ndhJ* | IGS | LSC |
| 46 | T | 10 | 10 | 47613 | 47622 | *rps4/trnT-UGU* | IGS | LSC |
| 47 | T | 10 | 10 | 48324 | 48333 | *trnT-UGU/trnL-UAA* | IGS | LSC |
| 48 | T | 10 | 10 | 65668 | 65677 | *petA/psbJ* | IGS | LSC |
| 49 | T | 10 | 10 | 73673 | 73682 | *clpP* | Intron | LSC |
| 50 | T | 11 | 11 | 7459 | 7469 | *trnQ-UUG/psbK* | IGS | LSC |
| 51 | T | 11 | 11 | 8590 | 8600 | *psbI/trnS-GCU* | IGS | LSC |
| 52 | T | 11 | 11 | 30261 | 30271 | *psbM/trnD-GUC* | IGS | LSC |
| 53 | T | 11 | 11 | 78117 | 78127 | *petB* | Intron | LSC |
| 54 | T | 11 | 11 | 73885 | 73895 | *clpP* | Intron | LSC |
| 55 | T | 11 | 11 | 117420 | 117430 | *rpl32* | pCDS | SSC |
| 56 | T | 12 | 12 | 31689 | 31700 | *trnD-GUC/trnY-GUA* | IGS | LSC |
| 57 | T | 12 | 12 | 116662 | 116673 | *ndhF/rpl32* | IGS | SSC |
| 58 | T | 12 | 12 | 72944 | 72955 | *clpP* | Intron | LSC |
| 59 | T | 12 | 12 | 84883 | 84894 | *rpl16* | Intron | LSC |
| 60 | T | 13 | 13 | 66977 | 66989 | *psbE/petL* | IGS | LSC |
| 61 | T | 13 | 13 | 54011 | 54023 | *ndhC/trnV-UAC* | IGS | LSC |
| 62* | T | 13 | 13 | 132742 | 132754 | *ycf1* | pCDS | IRA |
| 63 | T | 14 | 14 | 85036 | 85049 | *rpl16* | Intron | LSC |
| 64 | T | 16 | 16 | 83143 | 83158 | *rps8/rpl14* | IGS | LSC |
| 65 | T | 16 | 16 | 68998 | 69013 | *psaJ/rpl33* | IGS | LSC |
| 66 | T | 17 | 17 | 117761 | 117777 | *rpl32/trnL-UAG* | IGS | SSC |
| 67* | C | 10 | 10 | 41479 | 41488 | *psaA* | pCDS | LSC |
| 68* | G | 11 | 11 | 35487 | 35497 | *psbC* | pCDS | LSC |
| 69 | AT | 10 | 10 | 27411 | 27420 | *rpoB/trnC-GCA* | IGS | LSC |
| 70 | AT | 12 | 12 | 117640 | 117651 | *rpl32/trnL-UAG* | IGS | SSC |
| 71* | GA | 10 | 10 | 48872 | 48881 | *trnT-UGU/trnL-UAA* | IGS | LSC |
| 72* | TA | 10 | 10 | 73837 | 73846 | *clpP* | Intron | LSC |
| 73 | TA | 12 | 12 | 49903 | 49914 | *trnF-GAA/ndhJ* | IGS | LSC |
| 74* | TC | 10 | 10 | 126628 | 126637 | *ndhH* | pCDS | SSC |
| 75 | AGAA | 3 | 12 | 27443 | 27454 | *rpoB/trnC-GCA* | IGS | LSC |
| 76* | AGAA | 3 | 12 | 33757 | 33768 | *trnT-GGU/psbD* | IGS | LSC |
| 77 | ATAA | 3 | 12 | 52712 | 52723 | *ndhC/trnV-UAC* | IGS | LSC |
| 78* | ATTC | 3 | 12 | 77548 | 77559 | *petB* | Intron | LSC |
| 79 | TAAA | 3 | 12 | 52699 | 52710 | *ndhC/trnV-UAC* | IGS | LSC |
| 80 | AGATA | 3 | 15 | 14758 | 14772 | *atpH/atpI* | IGS | LSC |
| *E. lishihchenii* |  |  |  |  |  |  |  |  |
| 1 | A | 10 | 10 | 7974 | 7983 | *psbK/psbI* | IGS | LSC |
| 2 | A | 10 | 10 | 12987 | 12996 | *atpF* | Intron | LSC |
| 3 | A | 10 | 10 | 45832 | 45841 | *ycf3/trnS-GGA* | IGS | LSC |
| 4* | A | 10 | 10 | 66228 | 66237 | *psbF* | pCDS | LSC |
| 5 | A | 10 | 10 | 92030 | 92039 | *ycf2* | pCDS | IRB |
| 6 | A | 10 | 10 | 65267 | 65276 | *petA/psbJ* | IGS | LSC |
| 7 | A | 10 | 10 | 8779 | 8788 | *trnS-GCU/trnG-UCC* | IGS | LSC |
| 8 | A | 10 | 10 | 62160 | 62169 | *accD/psaI* | IGS | LSC |
| 9 | A | 10 | 10 | 77954 | 77963 | *petB* | Intron | LSC |
| 10 | A | 11 | 11 | 4014 | 4024 | *matK/trnK-UUU* | IGS | LSC |
| 11 | A | 11 | 11 | 13645 | 13655 | *atpF/atpH* | IGS | LSC |
| 12 | A | 11 | 11 | 28157 | 28167 | *rpoB/trnC-GCA* | IGS | LSC |
| 13 | A | 11 | 11 | 68249 | 68259 | *trnP-UGG/psaJ* | IGS | LSC |
| 14 | A | 11 | 11 | 68821 | 68831 | *psaJ/rpl33* | IGS | LSC |
| 15 | A | 11 | 11 | 82666 | 82676 | *rpl36/rps8* | IGS | LSC |
| 16 | A | 12 | 12 | 262 | 273 | *trnH-GUG/psbA* | IGS | LSC |
| 17 | A | 12 | 12 | 4843 | 4854 | *trnK-UUU/rps16* | IGS | LSC |
| 18 | A | 12 | 12 | 15025 | 15036 | *atpH/atpI* | IGS | LSC |
| 19 | A | 12 | 12 | 57542 | 57553 | *atpB/rbcL* | IGS | LSC |
| 20 | A | 12 | 12 | 117432 | 117443 | *ndhF/rpl32* | IGS | SSC |
| 21 | A | 13 | 13 | 72754 | 72766 | *clpP* | Intron | LSC |
| 22 | A | 13 | 13 | 125261 | 125273 | *ndhA* | Intron | SSC |
| 23 | A | 13 | 13 | 66857 | 66869 | *psbE/petL* | IGS | LSC |
| 24* | A | 13 | 13 | 112718 | 112730 | *ycf1* | pCDS | IRB |
| 25 | A | 13 | 13 | 131807 | 131819 | *ycf1* | pCDS | SSC |
| 26 | A | 13 | 13 | 72207 | 72219 | *rps12/clpP* | IGS | LSC |
| 27 | A | 14 | 14 | 84286 | 84299 | *rpl16* | Intron | LSC |
| 28 | A | 15 | 15 | 45572 | 45586 | *ycf3* | Intron | LSC |
| 29 | A | 15 | 15 | 61872 | 61886 | *accD/psaI* | IGS | LSC |
| 30 | T | 10 | 10 | 29513 | 29522 | *petN/psbM* | IGS | LSC |
| 31 | T | 10 | 10 | 30214 | 30223 | *psbM/trnD-GUC* | IGS | LSC |
| 32 | T | 10 | 10 | 5587 | 5596 | *trnK-UUU/rps16* | IGS | LSC |
| 33* | T | 10 | 10 | 18811 | 18820 | *rpoC2* | pCDS | LSC |
| 34* | T | 10 | 10 | 26599 | 26608 | *rpoB* | pCDS | LSC |
| 35 | T | 10 | 10 | 45504 | 45513 | *ycf3* | Intron | LSC |
| 36* | T | 10 | 10 | 54111 | 54120 | *trnV-UAC* | Intron | LSC |
| 37 | T | 10 | 10 | 154074 | 154083 | *ycf2* | pCDS | IRA |
| 38 | T | 10 | 10 | 43077 | 43086 | *psaA/ycf3* | IGS | LSC |
| 39 | T | 10 | 10 | 44483 | 44492 | *ycf3* | Intron | LSC |
| 40 | T | 10 | 10 | 71997 | 72006 | *rps12/clpP* | IGS | LSC |
| 41 | T | 10 | 10 | 77715 | 77724 | *petB* | Intron | LSC |
| 42 | T | 10 | 10 | 85440 | 85449 | rpl16/rps3 | IGS | LSC |
| 43 | T | 10 | 10 | 44009 | 44018 | *ycf3* | Intron | LSC |
| 44 | T | 10 | 10 | 65618 | 65627 | *petA/psbJ* | IGS | LSC |
| 45 | T | 10 | 10 | 73649 | 73658 | *clpP* | Intron | LSC |
| 46 | T | 11 | 11 | 6518 | 6528 | *rps16* | Intron | LSC |
| 47 | T | 11 | 11 | 7451 | 7461 | *trnQ-UUG/psbK* | IGS | LSC |
| 48 | T | 11 | 11 | 8577 | 8587 | *psbI/trnS-GCU* | IGS | LSC |
| 49 | T | 11 | 11 | 22950 | 22960 | *rpoC1* | Intron | LSC |
| 50 | T | 11 | 11 | 31640 | 31650 | *trnD-GUC/trnY-GUA* | IGS | LSC |
| 51 | T | 11 | 11 | 78087 | 78097 | *petB* | Intron | LSC |
| 52 | T | 11 | 11 | 128037 | 128047 | *rps15/ycf1* | IGS | SSC |
| 53 | T | 11 | 11 | 63212 | 63222 | *ycf4/cemA* | IGS | LSC |
| 54 | T | 11 | 11 | 73861 | 73871 | *clpP* | Intron | LSC |
| 55 | T | 11 | 11 | 118073 | 118083 | *rpl32* | pCDS | SSC |
| 56 | T | 11 | 11 | 50517 | 50527 | *trnF-GAA/ndhJ* | IGS | LSC |
| 57 | T | 11 | 11 | 84907 | 84917 | *rpl16* | Intron | LSC |
| 58 | T | 11 | 11 | 47586 | 47596 | *rps4/trnT-UGU* | IGS | LSC |
| 59 | T | 11 | 11 | 65411 | 65421 | *petA/psbJ* | IGS | LSC |
| 60 | T | 11 | 11 | 122830 | 122840 | *ndhE/ndhG* | IGS | SSC |
| 61 | T | 12 | 12 | 85161 | 85172 | *rpl16* | Intron | LSC |
| 62 | T | 12 | 12 | 74585 | 74596 | *clpP/psbB* | IGS | LSC |
| 63 | T | 12 | 12 | 53979 | 53990 | *ndhC/trnV-UAC* | IGS | LSC |
| 64 | T | 12 | 12 | 118423 | 118434 | *rpl32/trnL-UAG* | IGS | SSC |
| 65 | T | 13 | 13 | 83177 | 83189 | *rps8/rpl14* | IGS | LSC |
| 66 | T | 13 | 13 | 114294 | 114306 | *ycf1* | pCDS | SSC |
| 67* | T | 13 | 13 | 133383 | 133395 | *ycf1* | pCDS | IRA |
| 68 | T | 14 | 14 | 66942 | 66955 | *psbE/petL* | IGS | LSC |
| 69 | T | 14 | 14 | 117304 | 117317 | *ndhF/rpl32* | IGS | SSC |
| 70 | T | 14 | 14 | 85059 | 85072 | *rpl16* | Intron | LSC |
| 71 | T | 15 | 15 | 72917 | 72931 | *clpP* | Intron | LSC |
| 72 | T | 15 | 15 | 68966 | 68980 | *psaJ/rpl33* | IGS | LSC |
| 73* | C | 10 | 10 | 41433 | 41442 | *psaA* | pCDS | LSC |
| 74* | G | 11 | 11 | 35436 | 35446 | *psbC* | pCDS | LSC |
| 75 | AT | 5 | 10 | 27383 | 27392 | *rpoB/trnC-GCA* | IGS | LSC |
| 76 | AT | 8 | 16 | 118298 | 118313 | *rpl32/trnL-UAG* | IGS | SSC |
| 77* | GA | 5 | 10 | 48844 | 48853 | *trnT-UGU/trnL-UAA* | IGS | LSC |
| 78 | TA | 6 | 12 | 49875 | 49886 | *trnF-GAA/ndhJ* | IGS | LSC |
| 79* | TA | 5 | 10 | 73813 | 73822 | *clpP* | Intron | LSC |
| 80* | TC | 5 | 10 | 127268 | 127277 | *ndhH* | pCDS | SSC |
| 81 | CTT | 5 | 15 | 62092 | 62106 | *accD/psaI* | IGS | LSC |
| 82 | AGAA | 3 | 12 | 27415 | 27426 | *rpoB/trnC-GCA* | IGS | LSC |
| 83* | AGAA | 3 | 12 | 33706 | 33717 | *trnT-GGU/psbD* | IGS | LSC |
| 84 | AGAT | 3 | 12 | 78920 | 78931 | *petB/petD* | IGS | LSC |
| 85 | ATAA | 3 | 12 | 52682 | 52693 | *ndhC/trnV-UAC* | IGS | LSC |
| 86* | ATTC | 3 | 12 | 77518 | 77529 | *petB* | Intron | LSC |
| 87 | TTGA | 3 | 12 | 122354 | 122365 | *ndhE* | pCDS | SSC |
| *E. pseudowushanese* |  |  |  |  |  |  |  |  |
| 1 | A | 10 | 10 | 8028 | 8037 | *psbK/psbI* | IGS | LSC |
| 2 | A | 10 | 10 | 8576 | 8585 | *psbI/trnS-GCU* | IGS | LSC |
| 3 | A | 10 | 10 | 28189 | 28198 | *rpoB/trnC-GCA* | IGS | LSC |
| 4* | A | 10 | 10 | 66227 | 66236 | *psbF* | pCDS | LSC |
| 5 | A | 10 | 10 | 68226 | 68235 | *trnP-UGG/psaJ* | IGS | LSC |
| 6 | A | 10 | 10 | 92141 | 92150 | *ycf2* | pCDS | IRB |
| 7 | A | 10 | 10 | 65257 | 65266 | *petA/psbJ* | IGS | LSC |
| 8 | A | 10 | 10 | 116935 | 116944 | *ndhF/rpl32* | IGS | SSC |
| 9 | A | 10 | 10 | 66856 | 66865 | *psbE/petL* | IGS | LSC |
| 10 | A | 10 | 10 | 82776 | 82785 | *rpl36/rps8* | IGS | LSC |
| 11 | A | 10 | 10 | 78057 | 78066 | *petB* | Intron | LSC |
| 12 | A | 11 | 11 | 259 | 269 | *trnH-GUG/psbA* | IGS | LSC |
| 13 | A | 11 | 11 | 4021 | 4031 | *matK/trnK-UUU* | IGS | LSC |
| 14 | A | 11 | 11 | 4857 | 4867 | *trnK-UUU/rps16* | IGS | LSC |
| 15 | A | 11 | 11 | 13011 | 13021 | *atpF* | Intron | LSC |
| 16 | A | 11 | 11 | 13670 | 13680 | *atpF/atpH* | IGS | LSC |
| 17 | A | 11 | 11 | 45853 | 45863 | *ycf3/trnS-GGA* | IGS | LSC |
| 18 | A | 11 | 11 | 68797 | 68807 | *psaJ/rpl33* | IGS | LSC |
| 19 | A | 12 | 12 | 45596 | 45607 | *ycf3* | Intron | LSC |
| 20 | A | 13 | 13 | 15056 | 15068 | *atpH/atpI* | IGS | LSC |
| 21 | A | 13 | 13 | 57561 | 57573 | *atpB/rbcL* | IGS | LSC |
| 22 | A | 13 | 13 | 72844 | 72856 | *clpP* | Intron | LSC |
| 23 | A | 13 | 13 | 124762 | 124774 | *ndhA* | Intron | SSC |
| 24* | A | 13 | 13 | 112817 | 112829 | *ycf1* | pCDS | IRB |
| 25 | A | 13 | 13 | 131295 | 131307 | *ycf1* | pCDS | SSC |
| 26 | A | 13 | 13 | 8805 | 8817 | *trnS-GCU/trnG-UCC* | IGS | LSC |
| 27 | A | 13 | 13 | 72297 | 72309 | *rps12/clpP* | IGS | LSC |
| 28 | A | 14 | 14 | 84395 | 84408 | *rpl16* | Intron | LSC |
| 29 | A | 15 | 15 | 38488 | 38502 | *rps14/psaB* | IGS | LSC |
| 30 | A | 20 | 20 | 61856 | 61875 | *accD/psaI* | IGS | LSC |
| 31 | T | 10 | 10 | 7464 | 7473 | *trnQ-UUG/psbK* | IGS | LSC |
| 32 | T | 10 | 10 | 29548 | 29557 | *petN/psbM* | IGS | LSC |
| 33 | T | 10 | 10 | 30249 | 30258 | *psbM/trnD-GUC* | IGS | LSC |
| 34 | T | 10 | 10 | 5600 | 5609 | *trnK-UUU/rps16* | IGS | LSC |
| 35* | T | 10 | 10 | 18843 | 18852 | *rpoC2* | pCDS | LSC |
| 36* | T | 10 | 10 | 26631 | 26640 | *rpoB* | pCDS | LSC |
| 37* | T | 10 | 10 | 54130 | 54139 | *trnV-UAC* | Intron | LSC |
| 38 | T | 10 | 10 | 153550 | 153559 | *ycf2* | pCDS | IRA |
| 39 | T | 10 | 10 | 85551 | 85560 | *rpl16/rps3* | IGS | LSC |
| 40 | T | 10 | 10 | 117574 | 117583 | *rpl32* | pCDS | SSC |
| 41 | T | 10 | 10 | 44032 | 44041 | *ycf3* | Intron | LSC |
| 42 | T | 10 | 10 | 47608 | 47617 | *rps4/trnT-UGU* | IGS | LSC |
| 43 | T | 10 | 10 | 65409 | 65418 | *petA/psbJ* | IGS | LSC |
| 44 | T | 10 | 10 | 82736 | 82745 | *rpl36/rps8* | pCDS | LSC |
| 45 | T | 11 | 11 | 6531 | 6541 | *rps16* | Intron | LSC |
| 46 | T | 11 | 11 | 22982 | 22992 | *rpoC1* | Intron | LSC |
| 47 | T | 11 | 11 | 43104 | 43114 | *psaA/ycf3* | IGS | LSC |
| 48 | T | 11 | 11 | 73957 | 73967 | *clpP* | Intron | LSC |
| 49 | T | 11 | 11 | 71974 | 71984 | *rps12/clpP* | IGS | LSC |
| 50 | T | 11 | 11 | 77817 | 77827 | *petB* | Intron | LSC |
| 51 | T | 11 | 11 | 50531 | 50541 | *trnF-GAA/ndhJ* | IGS | LSC |
| 52 | T | 11 | 11 | 73744 | 73754 | *clpP* | Intron | LSC |
| 53 | T | 12 | 12 | 8602 | 8613 | *psbI/trnS-GCU* | IGS | LSC |
| 54 | T | 12 | 12 | 31676 | 31687 | *trnD-GUC/trnY-GUA* | IGS | LSC |
| 55 | T | 12 | 12 | 45526 | 45537 | *ycf3* | Intron | LSC |
| 56 | T | 12 | 12 | 78190 | 78201 | *petB* | Intron | LSC |
| 57 | T | 12 | 12 | 85272 | 85283 | *rpl16* | Intron | LSC |
| 58 | T | 12 | 12 | 63201 | 63212 | *ycf4/cemA* | IGS | LSC |
| 59 | T | 12 | 12 | 85016 | 85027 | *rpl16* | Intron | LSC |
| 60 | T | 12 | 12 | 65615 | 65626 | *petA/psbJ* | IGS | LSC |
| 61 | T | 13 | 13 | 53997 | 54009 | *ndhC/trnV-UAC* | IGS | LSC |
| 62* | T | 13 | 13 | 132871 | 132883 | *ycf1* | pCDS | IRA |
| 63 | T | 14 | 14 | 116807 | 116820 | *ndhF/rpl32* | IGS | SSC |
| 64 | T | 14 | 14 | 73007 | 73020 | *clpP* | Intron | LSC |
| 65 | T | 14 | 14 | 83286 | 83299 | *rps8/rpl14* | IGS | LSC |
| 66 | T | 15 | 15 | 66925 | 66939 | *psbE/petL* | IGS | LSC |
| 67 | T | 15 | 15 | 85169 | 85183 | *rpl16* | Intron | LSC |
| 68 | T | 15 | 15 | 117923 | 117937 | *rpl32/trnL-UAG* | IGS | SSC |
| 69 | T | 16 | 16 | 68942 | 68957 | *psaJ/rpl33* | IGS | LSC |
| 70* | C | 10 | 10 | 41460 | 41469 | *psaA* | pCDS | LSC |
| 71* | G | 11 | 11 | 35474 | 35484 | *psbC* | pCDS | LSC |
| 72 | AT | 5 | 10 | 27415 | 27424 | *rpoB/trnC-GCA* | IGS | LSC |
| 73 | AT | 7 | 14 | 117793 | 117806 | *rpl32/trnL-UAG* | IGS | SSC |
| 74* | GA | 5 | 10 | 48865 | 48874 | *trnT-UGU/trnL-UAA* | IGS | LSC |
| 75* | TA | 5 | 10 | 73909 | 73918 | *clpP* | Intron | LSC |
| 76 | TA | 6 | 12 | 49896 | 49907 | *trnF-GAA/ndhJ* | IGS | LSC |
| 77* | TC | 5 | 10 | 126776 | 126785 | *ndhH* | pCDS | SSC |
| 78 | AGAA | 3 | 12 | 27447 | 27458 | *rpoB/trnC-GCA* | IGS | LSC |
| 79* | AGAA | 3 | 12 | 33744 | 33755 | *trnT-GGU/psbD* | IGS | LSC |
| 80 | ATAA | 3 | 12 | 52700 | 52711 | *ndhC/trnV-UAC* | IGS | LSC |
| 81* | ATTC | 3 | 12 | 77620 | 77631 | *petB* | Intron | LSC |
| 82 | TAAA | 3 | 12 | 52687 | 52698 | *ndhC/trnV-UAC* | IGS | LSC |
| 83 | TTGA | 3 | 12 | 121857 | 121868 | *ndhE* | pCDS | SSC |
| 84 | AGATA | 3 | 15 | 14767 | 14781 | *atpH/atpI* | IGS | LSC |
| *E. koreanum* |  |  |  |  |  |  |  |  |
| 1 | A | 10 | 10 | 38875 | 38884 | *rps14/psaB* | IGS | LSC |
| 2 | A | 10 | 10 | 46252 | 46261 | *ycf3/trnS-GGA* | IGS | LSC |
| 3 | A | 10 | 10 | 57984 | 57993 | *atpB/rbcL* | IGS | LSC |
| 4* | A | 10 | 10 | 66677 | 66686 | *psbF* | pCDS | LSC |
| 5 | A | 10 | 10 | 68666 | 68675 | *trnP-UGG/psaJ* | IGS | LSC |
| 6 | A | 10 | 10 | 85970 | 85979 | *rpl16* | Intron | LSC |
| 7 | A | 10 | 10 | 92680 | 92689 | *ycf2* | pCDS | IRB |
| 8 | A | 11 | 11 | 45938 | 45948 | *ycf3* | Intron | LSC |
| 9 | A | 11 | 11 | 45996 | 46006 | *ycf3* | Intron | LSC |
| 10 | A | 11 | 11 | 69237 | 69247 | *psaJ/rpl33* | IGS | LSC |
| 11 | A | 11 | 11 | 73341 | 73351 | *clpP* | Intron | LSC |
| 12 | A | 11 | 11 | 125268 | 125278 | *ndhA* | Intron | SSC |
| 13 | A | 12 | 12 | 4870 | 4881 | *trnK-UUU/rps16* | IGS | LSC |
| 14 | A | 12 | 12 | 28286 | 28297 | *rpoB/trnC-GCA* | IGS | LSC |
| 15 | A | 12 | 12 | 28412 | 28423 | *rpoB/trnC-GCA* | IGS | LSC |
| 16 | A | 12 | 12 | 65714 | 65725 | *petA/psbJ* | IGS | LSC |
| 17 | A | 12 | 12 | 117462 | 117473 | *ndhF/rpl32* | IGS | SSC |
| 18 | A | 12 | 12 | 125407 | 125418 | *ndhA* | Intron | SSC |
| 19 | A | 13 | 13 | 13105 | 13117 | *atpF* | Intron | LSC |
| 20 | A | 13 | 13 | 67306 | 67318 | *psbE/petL* | IGS | LSC |
| 21 | A | 13 | 13 | 83253 | 83265 | *rpl36/rps8* | IGS | LSC |
| 22* | A | 13 | 13 | 113362 | 113374 | *ycf1* | pCDS | IRB |
| 23 | A | 13 | 13 | 131853 | 131865 | *ycf1* | pCDS | SSC |
| 24 | A | 14 | 14 | 8672 | 8685 | *psbI/trnS-GCU* | IGS | LSC |
| 25 | A | 14 | 14 | 15142 | 15155 | *atpH/atpI* | IGS | LSC |
| 26 | A | 14 | 14 | 62306 | 62319 | *accD/psaI* | IGS | LSC |
| 27 | A | 15 | 15 | 4035 | 4049 | *matK/trnK-UUU* | IGS | LSC |
| 28 | A | 15 | 15 | 8901 | 8915 | *trnS-GCU/trnG-UCC* | IGS | LSC |
| 29 | A | 15 | 15 | 72768 | 72782 | *rps12/clpP* | IGS | LSC |
| 30 | A | 17 | 17 | 44087 | 44103 | *psaA/ycf3* | IGS | LSC |
| 31 | T | 10 | 10 | 6549 | 6558 | *rps16* | Intron | LSC |
| 32 | T | 10 | 10 | 13368 | 13377 | *atpF/atpH* | IGS | LSC |
| 33 | T | 10 | 10 | 23078 | 23087 | *rpoC1* | Intron | LSC |
| 34 | T | 10 | 10 | 29927 | 29936 | *petN/psbM* | IGS | LSC |
| 35 | T | 10 | 10 | 30636 | 30645 | *psbM/trnD-GUC* | IGS | LSC |
| 36 | T | 10 | 10 | 37287 | 37296 | *trnS-UGA/psbZ* | IGS | LSC |
| 37 | T | 10 | 10 | 10152 | 10161 | *trnG-UCC/trnR-UCU* | IGS | LSC |
| 38* | T | 10 | 10 | 18938 | 18947 | *rpoC2* | pCDS | LSC |
| 39* | T | 10 | 10 | 26726 | 26735 | *rpoB* | pCDS | LSC |
| 40 | T | 10 | 10 | 45927 | 45936 | *ycf3* | Intron | LSC |
| 41* | T | 10 | 10 | 54535 | 54544 | trnV-UAC | Intron | LSC |
| 42 | T | 10 | 10 | 78672 | 78681 | *petB* | Intron | LSC |
| 43 | T | 10 | 10 | 83242 | 83251 | *rpl36/rps8* | IGS | LSC |
| 44 | T | 10 | 10 | 117326 | 117335 | *ndhF/rpl32* | IGS | SSC |
| 45 | T | 10 | 10 | 128027 | 128036 | *rps15/ycf1* | IGS | SSC |
| 46 | T | 10 | 10 | 154090 | 154099 | *ycf2* | pCDS | IRA |
| 47 | T | 11 | 11 | 32062 | 32072 | *trnD-GUC/trnY-GUA* | IGS | LSC |
| 48 | T | 11 | 11 | 43486 | 43496 | *psaA/ycf3* | IGS | LSC |
| 49 | T | 11 | 11 | 44898 | 44908 | *ycf3* | Intron | LSC |
| 50 | T | 11 | 11 | 63654 | 63664 | *ycf4/cemA* | IGS | LSC |
| 51 | T | 11 | 11 | 74447 | 74457 | *clpP* | Intron | LSC |
| 52 | T | 12 | 12 | 7561 | 7572 | *trnQ-UUG/psbK* | IGS | LSC |
| 53 | T | 12 | 12 | 8698 | 8709 | *psbI/trnS-GCU* | IGS | LSC |
| 54 | T | 12 | 12 | 7474 | 7485 | *trnQ-UUG/psbK* | IGS | LSC |
| 55 | T | 12 | 12 | 72423 | 72434 | *rps12/clpP* | IGS | LSC |
| 56 | T | 12 | 12 | 73508 | 73519 | *clpP* | Intron | LSC |
| 57 | T | 12 | 12 | 78299 | 78310 | *petB* | Intron | LSC |
| 58 | T | 12 | 12 | 83765 | 83776 | *rps8/rpl14* | IGS | LSC |
| 59 | T | 12 | 12 | 86088 | 86099 | *rpl16/rps3* | IGS | LSC |
| 60 | T | 11 | 11 | 118086 | 118096 | *rpl32* | pCDS | SSC |
| 61 | T | 13 | 13 | 44421 | 44433 | *ycf3* | Intron | LSC |
| 62 | T | 13 | 13 | 46866 | 46878 | *ycf3/trnS-GGA* | IGS | LSC |
| 63 | T | 13 | 13 | 54403 | 54415 | *ndhC/trnV-UAC* | IGS | LSC |
| 64 | T | 13 | 13 | 83802 | 83814 | *rps8/rpl14* | IGS | LSC |
| 65 | T | 13 | 13 | 85689 | 85701 | *rpl16* | Intron | LSC |
| 66* | T | 13 | 13 | 133405 | 133417 | *ycf1* | pCDS | IRA |
| 67 | T | 14 | 14 | 50946 | 50959 | *trnF-GAA/ndhJ* | IGS | LSC |
| 68 | T | 14 | 14 | 85534 | 85547 | *rpl16* | Intron | LSC |
| 69 | T | 14 | 14 | 118421 | 118434 | *rpl32/trnL-UAG* | IGS | SSC |
| 70 | T | 15 | 15 | 69381 | 69395 | *psaJ/rpl33* | IGS | LSC |
| 71* | C | 10 | 10 | 41842 | 41851 | *psaA* | pCDS | LSC |
| 72* | G | 11 | 11 | 35855 | 35865 | *psbC* | pCDS | LSC |
| 73 | AT | 7 | 14 | 85797 | 85810 | *rpl16* | Intron | LSC |
| 74 | AT | 7 | 14 | 118304 | 118317 | *rpl32/trnL-UAG* | IGS | SSC |
| 75 | AT | 7 | 16 | 27510 | 27525 | *rpoB/trnC-GCA* | IGS | LSC |
| 76* | GA | 5 | 10 | 49272 | 49281 | *trnT-UGU/trnL-UAA* | IGS | LSC |
| 77 | TA | 5 | 10 | 50303 | 50312 | *trnF-GAA/ndhJ* | IGS | LSC |
| 78* | TA | 5 | 10 | 74399 | 74408 | *clpP* | Intron | LSC |
| 79* | TC | 5 | 10 | 127258 | 127267 | *ndhH* | pCDS | SSC |
| 80* | AGAA | 3 | 12 | 34125 | 34136 | *trnT-GGU/psbD* | IGS | LSC |
| 81 | TAAA | 4 | 16 | 53103 | 53118 | *ndhC/trnV-UAC* | IGS | LSC |
| 82* | ATTC | 3 | 12 | 78102 | 78113 | *petB* | Intron | LSC |
| 83 | TCTT | 3 | 12 | 10207 | 10218 | *trnG-UCC/trnR-UCU* | IGS | LSC |
| 84 | TTGA | 3 | 12 | 122374 | 122385 | *ndhE* | pCDS | SSC |
| 85 | TTTCTA | 3 | 18 | 73440 | 73457 | *clpP* | Intron | LSC |
