# Supplementary material for: The Complete Chloroplast Genome Sequences of Five Epimedium Species: Lights into Phylogenetic and Taxonomic Analyses
Source: Front Plant Sci. 2016 Mar 15;7:306. doi: 10.3389/fpls.2016.00306 (PMC4791396; doi:10.3389/fpls.2016.00306)
Supplement: Supplementary file 7 [file Table7.DOCX]

Table S7. The number of different repeat units in the five *Epimedium* cp genomes. P1 to P6 represented SSR loci with mono-, di-, tri-, tetra-, penta-, and hexanucleotide repeats, respectively.

| **Species** | **P1** | | | | **P2** | | | | **P3** | **P4** | | | | | | | **P5** | **P6** | | | **Total** |
| --- | --- | --- | --- | --- | --- | --- | --- | --- | --- | --- | --- | --- | --- | --- | --- | --- | --- | --- | --- | --- | --- |
|  | **A** | **T** | **C** | **G** | **AT** | **GA** | **TA** | **TC** | **CTT** | **AGAA** | **AGAT** | **ATAA** | **ATTC** | **TAAA** | **TCTT** | **TTGA** | **AGATA** | **ATCAAT** | **GATATT** | **TTTCTA** |  |
| *E.acuminatum* | 34 | 36 | 1 | 2 | 2 | 1 | 2 | 1 | / | 2 | / | / | 1 | 1 | / | 1 | 1 | 1 | 1 | / | 87 |
| *E.dolichostemon* | 29 | 37 | 1 | 1 | 2 | 1 | 2 | 1 | / | 2 | / | 1 | 1 | 1 | / | / | 1 | / | / | / | 80 |
| *E.lishihchenii* | 29 | 43 | 1 | 1 | 2 | 1 | 2 | 1 | 1 | 2 | 1 | 1 | 1 | / | / | 1 | / | / | / | / | 87 |
| *E.pseudowushanense* | 30 | 39 | 1 | 1 | 2 | 1 | 2 | 1 | / | 2 | / | 1 | 1 | 1 | / | 1 | 1 | / | / | / | 84 |
| *E.koreanum* | 30 | 40 | 1 | 1 | 3 | 1 | 2 | 1 | / | 1 | / | / | 1 | 1 | 1 | 1 | / | / | / | 1 | 85 |
| **Total** | 41 | 52 | 1 | 2 | 3 | 1 | 2 | 1 | 1 | 2 | 1 | 1 | 1 | 1 | 1 | 1 | 1 | 1 | 1 | 1 | 116 |
|  | 96 | | | | 7 | | | | 1 | 8 | | | | | | | 1 | 3 | | |  |
