# Supplementary material for: The Complete Chloroplast Genome Sequences of Five Epimedium Species: Lights into Phylogenetic and Taxonomic Analyses
Source: Front Plant Sci. 2016 Mar 15;7:306. doi: 10.3389/fpls.2016.00306 (PMC4791396; doi:10.3389/fpls.2016.00306)
Supplement: Supplementary file 8 [file Table8.DOCX]

Table S8. A list of repeated sequences and their locations identified in the five *Epimedium* chloroplast genomes. ACUM, DOLI, LISH, PSEU, KORE represented *E. acuminatum*, *E. dolichostemon*, *E. lishihchenii*, *E. pseudowushanense*, and *E. koreanum*, respectively.

| Genomes /SSR No. | Repeat Type | Length  (bp) | PositionA | Locus | Region | Position B | Locus | Region | Shared genomes |
| --- | --- | --- | --- | --- | --- | --- | --- | --- | --- |
| ACUM |  |  |  |  |  |  |  |  |  |
| 1 | P | 131 | 5852 | *trnK-UUU-rps16, rps16* | LSC | 103576 | *trnV-GAC-rrn16, rrn16* | IRA | ACUM, DOLI, LISH, PSEU |
| 2 | F | 131 | 5852 | *trnK-UUU-rps16, rps16* | LSC | 141966 | *rrn16, rrn16-trnV-GAC* | IRB | ACUM, DOLI, LISH, PSEU |
| 3 | F | 107 | 4648 | *trnK-UUU-rps16* | LSC | 109577 | *rrn23* | IRA | ALL |
| 4 | P | 107 | 4648 | *trnK-UUU-rps16* | LSC | 135989 | *rrn23* | IRB | ALL |
| 5 | F | 82 | 113965 | *ycf1* | IRA | 114100 | *ycf1* | IRA | ACUM, DOLI, LISH, PSEU |
| 6 | P | 82 | 113965 | *ycf1* | IRA | 131491 | *ycf1* | IRB | ACUM, DOLI, LISH, PSEU |
| 7 | P | 82 | 114100 | *ycf1* | IRA | 131626 | *ycf1* | IRB | ACUM, DOLI, LISH, PSEU |
| 8 | F | 82 | 131491 | *ycf1* | IRB | 131626 | *ycf1* | IRB | ACUM, DOLI, LISH, PSEU |
| 9 | P | 71 | 62245 | *accD-psaI* | LSC | 62245 | *accD-psaI* | LSC | ALL |
| 10 | F | 77 | 4758 | *trnK-UUU-rps16* | LSC | 109689 | *rrn23* | IRA | ALL |
| 11 | P | 77 | 4758 | *trnK-UUU-rps16* | LSC | 135907 | *rrn23* | IRB | ALL |
| 12 | F | 69 | 5522 | *trnK-UUU-rps16* | LSC | 5673 | *trnK-UUU-rps16* | LSC | ACUM |
| 13 | F | 62 | 131511 | *ycf1* | IRB | 131646 | *ycf1* | IRB | ACUM, DOLI, LISH, PSEU |
| 14 | F | 53 | 4702 | *trnK-UUU-rps16* | LSC | 109631 | *rrn23* | IRA | ACUM, DOLI, PSEU |
| 15 | P | 53 | 4702 | *trnK-UUU-rps16* | LSC | 135989 | *rrn23* | IRB | ACUM, DOLI, PSEU |
| 16 | P | 48 | 54791 | *trnV-UAC-trnM-CAU* | LSC | 54791 | *trnV-UAC-trnM-CAU* | LSC | ALL |
| 17 | F | 49 | 4786 | *trnK-UUU-rps16* | LSC | 109717 | *rrn23* | IRA | ACUM, DOLI, LISH, PSEU |
| 18 | P | 49 | 4786 | *trnK-UUU-rps16* | LSC | 135907 | *rrn23* | IRB | ACUM, DOLI, LISH, PSEU |
| 19 | P | 49 | 98000 | *trnL-CAA-ndhB* | IRA | 98000 | *trnL-CAA-ndhB* | IRA | ALL |
| 20 | F | 49 | 98000 | *trnL-CAA-ndhB* | IRA | 147624 | *ndhB-trnL-CAA* | IRB | ALL |
| 21 | P | 49 | 147624 | *ndhB-trnL-CAA* | IRB | 147624 | *ndhB-trnL-CAA* | IRB | ALL |
| 22 | P | 44 | 30016 | *petN-psbM/* | LSC | 30016 | *petN-psbM* | LSC | ALL |
| 23 | F | 50 | 74410 | *clpP_extron_2-clpP_extron_1* | LSC | 74458 | *clpP_extron_2-clpP_extron_1* | LSC | ALL |
| 24 | F | 51 | 72222 | *rps12-clpP* | LSC | 72267 | *rps12-clpP* | LSC | ACUM |
| 25 | F | 39 | 113963 | *ycf1* | IRA | 114029 | *ycf1* | IRA | ACUM |
| 26 | P | 39 | 113963 | *ycf1* | IRA | 131605 | *ycf1* | IRB | ACUM |
| 27 | P | 39 | 114029 | *ycf1* | IRA | 131671 | *ycf1* | IRB | ACUM |
| 28 | F | 39 | 131605 | *ycf1* | IRB | 131671 | *ycf1* | IRB | ACUM |
| 29 | P | 35 | 5948 | *trnK-UUU-rps16, rps16* | LSC | 103576 | *trnV-GAC-rrn16* | IRA | ACUM, DOLI, LISH, PSEU |
| 30 | F | 35 | 5948 | *trnK-UUU-rps16, rps16* | LSC | 142062 | *rrn16-trnV-GAC* | IRB | ACUM, DOLI, LISH, PSEU |
| 31 | P | 35 | 65768 | *petA-psbJ* | LSC | 65810 | *petA-psbJ* | LSC | ACUM, DOLI, LISH, PSEU |
| 32 | P | 43 | 49737 | *trnL-UAA-trnF-GAA* | LSC | 49737 | *trnL-UAA-trnF-GAA* | LSC | ACUM, DOLI, LISH, PSEU |
| 33 | F | 43 | 113881 | *ycf1* | IRA | 113893 | *ycf1* | IRA | ALL |
| 34 | P | 43 | 113881 | *ycf1* | IRA | 131737 | *ycf1* | IRB | ALL |
| 35 | P | 43 | 113893 | *ycf1* | IRA | 131749 | *ycf1* | IRB | ALL |
| 36 | F | 43 | 131737 | *ycf1* | IRB | 131749 | *ycf1* | IRB | ACUM, DOLI, LISH, PSEU |
| 37 | F | 37 | 113979 | *ycf1* | IRA | 114066 | *ycf1* | IRA | ACUM |
| 38 | P | 37 | 113979 | *ycf1* | IRA | 131570 | *ycf1* | IRB | ACUM |
| 39 | F | 37 | 114066 | *ycf1* | IRA | 114114 | *ycf1* | IRA | ACUM |
| 40 | P | 37 | 114066 | *ycf1* | IRA | 131522 | *ycf1* | IRB | ACUM |
| 41 | P | 37 | 114066 | *ycf1* | IRA | 131657 | *ycf1* | IRB | ACUM |
| 42 | P | 37 | 114114 | *ycf1* | IRA | 131570 | *ycf1* | IRB | ACUM |
| 43 | F | 37 | 131522 | *ycf1* | IRB | 131570 | *ycf1* | IRB | ACUM |
| 44 | F | 37 | 131570 | *ycf1* | IRB | 131657 | *ycf1* | IRB | ACUM |
| 45 | F | 39 | 45000 | *ycf3_extron_2-ycf3_extron_1* | LSC | 102017 | *rps12-trnV-GAC* | IRA | ALL |
| 46 | P | 39 | 45000 | *ycf3_extron_2-ycf3_extron_1* | LSC | 143617 | *trnV-GAC-rps12* | IRB | ALL |
| 47 | P | 36 | 8677 | *psbI-trnS-GCU, trnS-GCU* | LSC | 46735 | *trnS-GGA, trnS-GGA-rps4* | LSC | ACUM, DOLI, LISH, PSEU |
| 48 | F | 32 | 114036 | *ycf1* | IRA | 114105 | *ycf1* | IRB | ACUM |
| 49 | P | 32 | 114036 | *ycf1* | IRA | 131536 | *ycf1* | IRB | ACUM |
| DOLI |  |  |  |  |  |  |  |  |  |
| 1 | P | 131 | 5833 | *trnK-UUU-rps16, rps16* | LSC | 103457 | *trnV-GAC-rrn16, rrn16* | IRA | ACUM, DOLI, LISH, PSEU |
| 2 | F | 131 | 5833 | *trnK-UUU-rps16, rps16* | LSC | 141845 | *rrn16, rrn16-trnV-GAC* | IRB | ACUM, DOLI, LISH, PSEU |
| 3 | F | 107 | 4641 | *trnK-UUU-rps16* | LSC | 109459 | *rrn23* | IRA | ALL |
| 4 | P | 107 | 4641 | *trnK-UUU-rps16* | LSC | 135867 | *rrn23* | IRB | ALL |
| 5 | F | 82 | 113862 | *ycf1* | IRA | 113976 | *ycf1* | IRA | ACUM, DOLI, LISH, PSEU |
| 6 | P | 82 | 113862 | *ycf1* | IRA | 131375 | *ycf1* | IRA | ACUM, DOLI, LISH, PSEU |
| 7 | P | 82 | 113976 | *ycf1* | IRA | 131489 | *ycf1* | IRA | ACUM, DOLI, LISH, PSEU |
| 8 | F | 82 | 131375 | *ycf1* | IRA | 131489 | *ycf1* | IRA | ACUM, DOLI, LISH, PSEU |
| 9 | P | 71 | 62202 | *accD-psaI* | LSC | 62202 | *accD-psaI* | LSC | ALL |
| 10 | F | 77 | 4751 | *trnK-UUU-rps16* | LSC | 109571 | *rrn23* | IRA | ALL |
| 11 | P | 77 | 4751 | *trnK-UUU-rps16* | LSC | 135785 | *rrn23* | IRB | ALL |
| 12 | F | 62 | 131395 | *ycf1* | IRB | 131509 | *ycf1* | IRB | ACUM, DOLI, LISH, PSEU |
| 13 | F | 53 | 4695 | *trnK-UUU-rps16* | LSC | 109513 | *rrn23* | IRA | ACUM, DOLI, PSEU |
| 14 | P | 53 | 4695 | *trnK-UUU-rps16* | LSC | 135867 | *rrn23* | IRB | ACUM, DOLI, PSEU |
| 15 | P | 48 | 54732 | *trnV-UAC-trnM-CAU* | LSC | 54732 | *trnV-UAC-trnM-CAU* | LSC | ALL |
| 16 | F | 53 | 113860 | *ycf1* | IRA | 113926 | *ycf1* | IRA | DOLI, LISH, PSEU, KORE |
| 17 | P | 53 | 113860 | *ycf1* | IRA | 131454 | *ycf1* | IRB | DOLI, LISH, PSEU, KORE |
| 18 | P | 53 | 113926 | *ycf1* | IRA | 131520 | *ycf1* | IRB | DOLI, LISH, PSEU, KORE |
| 19 | F | 53 | 131454 | *ycf1* | IRB | 131520 | *ycf1* | IRB | DOLI, LISH, PSEU, KORE |
| 20 | F | 49 | 4779 | *trnK-UUU-rps16* | LSC | 109599 | *rrn23* | IRA | ACUM, DOLI, LISH, PSEU |
| 21 | P | 49 | 4779 | *trnK-UUU-rps16* | LSC | 135785 | *rrn23* | IRB | ACUM, DOLI, LISH, PSEU |
| 22 | P | 49 | 97881 | *trnL-CAA-ndhB* | IRA | 97881 | *trnL-CAA-ndhB* | IRA | ALL |
| 23 | F | 49 | 97881 | *trnL-CAA-ndhB* | IRA | 147503 | *ndhB-trnL-CAA* | IRB | ALL |
| 24 | P | 49 | 147503 | *ndhB-trnL-CAA* | IRB | 147503 | *ndhB-trnL-CAA* | IRB | ALL |
| 25 | P | 44 | 29974 | *petN-psbM* | LSC | 29974 | *petN-psbM* | LSC | ALL |
| 26 | F | 50 | 74304 | *clpP_extron_2-clpP_extron_1* | LSC | 74352 | *clpP_extron_2-clpP_extron_1* | LSC | ALL |
| 27 | F | 46 | 113933 | *ycf1* | IRA | 113981 | *ycf1* | IRA | DOLI, LISH, PSEU, KORE |
| 28 | P | 46 | 113933 | *ycf1* | IRA | 131406 | *ycf1* | IRB | DOLI, LISH, PSEU, KORE |
| 29 | P | 46 | 113981 | *ycf1* | IRA | 131454 | *ycf1* | IRB | DOLI, LISH, PSEU, KORE |
| 30 | F | 46 | 131406 | *ycf1* | IRB | 131454 | *ycf1* | IRB | DOLI, LISH, PSEU, KORE |
| 31 | F | 37 | 91955 | *ycf2* | IRA | 92012 | *ycf2* | IRA | DOLI, LISH, PSEU, KORE |
| 32 | P | 37 | 91955 | *ycf2* | IRA | 153384 | *ycf2* | IRB | DOLI, LISH, PSEU, KORE |
| 33 | P | 37 | 92012 | *ycf2* | IRA | 153441 | *ycf2* | IRB | DOLI, LISH, PSEU, KORE |
| 34 | F | 37 | 153384 | *ycf2* | IRB | 153441 | *ycf2* | IRB | DOLI, LISH, PSEU, KORE |
| 35 | F | 40 | 131467 | *ycf1* | IRB | 131533 | *ycf1* | IRB | DOLI, LISH, PSEU, KORE |
| 36 | P | 36 | 36466 | *psbC-trnS-UGA* | LSC | 36514 | *psbC-trnS-UGA* | LSC | DOLI |
| 37 | P | 35 | 5929 | *trnK-UUU-rps16, rps16* | LSC | 103457 | *trnV-GAC-rrn16* | IRA | ACUM, DOLI, LISH, PSEU |
| 38 | F | 35 | 5929 | *trnK-UUU-rps16, rps16* | LSC | 141941 | *rrn16-trnV-GAC* | IRB | ACUM, DOLI, LISH, PSEU |
| 39 | P | 35 | 65740 | *petA-psbJ* | LSC | 65782 | *petA-psbJ* | LSC | ACUM, DOLI, LISH, PSEU |
| 40 | P | 43 | 49670 | *trnL-UAA-trnF-GAA* | LSC | 49670 | *trnL-UAA-trnF-GAA* | LSC | ACUM, DOLI, LISH, PSEU |
| 41 | F | 43 | 113778 | *ycf1* | IRA | 113790 | *ycf1* | IRA | ALL |
| 42 | P | 43 | 113778 | *ycf1* | IRA | 131600 | *ycf1* | IRB | ALL |
| 43 | P | 43 | 113790 | *ycf1* | IRA | 131612 | *ycf1* | IRB | ALL |
| 44 | F | 43 | 131600 | *ycf1* | IRB | 131612 | *ycf1* | IRB | ACUM, DOLI, LISH, PSEU |
| 45 | F | 33 | 131419 | *ycf1* | IRB | 131467 | *ycf1* | IRB | DOLI, LISH, PSEU |
| 46 | F | 39 | 44937 | *ycf3_extron_2-ycf3_extron_1* | LSC | 101898 | *rps12-trnV-GAC* | IRA | ALL |
| 47 | P | 39 | 44937 | *ycf3_extron_2-ycf3_extron_1* | LSC | 143496 | *trnV-GAC-rps12* | IRB | ALL |
| 48 | P | 36 | 8634 | *psbI-trnS-GCU, trnS-GCU* | LSC | 46670 | *trnS-GGA, trnS-GGA-rps4* | LSC | ACUM, DOLI, LISH, PSEU |
| 49 | F | 32 | 5509 | *trnK-UUU-rps16* | LSC | 5659 | *trnK-UUU-rps16* | LSC | DOLI, LISH |
| LISH |  |  |  |  |  |  |  |  |  |
| 1 | P | 5816 | 131 | *trnK-UUU-rps16, rps16* | LSC | 103495 | *trnV-GAC-rrn16, rrn16* | IRA | ACUM, DOLI, LISH, PSEU |
| 2 | F | 5816 | 131 | *trnK-UUU-rps16, rps16* | LSC | 142486 | *rrn16, rrn16-trnV-GAC* | IRB | ACUM, DOLI, LISH, PSEU |
| 3 | F | 4623 | 107 | *trnK-UUU-rps16* | LSC | 109497 | *rrn23* | IRA | ALL |
| 4 | P | 4623 | 107 | *trnK-UUU-rps16* | LSC | 136508 | *rrn23* | IRB | ALL |
| 5 | F | 4634 | 96 | *trnK-UUU-rps16* | LSC | 109508 | *rrn23* | IRA | LSIH |
| 6 | P | 4634 | 96 | *trnK-UUU-rps16* | LSC | 136508 | *rrn23* | IRB | LISH |
| 7 | F | 113900 | 82 | *ycf1* | IRA | 114014 | *ycf1* | IRA | ACUM, DOLI, LISH, PSEU |
| 8 | P | 113900 | 82 | *ycf1* | IRA | 132016 | *ycf1* | IRA | ACUM, DOLI, LISH, PSEU |
| 9 | P | 114014 | 82 | *ycf1* | IRA | 132130 | *ycf1* | IRA | ACUM, DOLI, LISH, PSEU |
| 10 | F | 132016 | 82 | *ycf1* | IRA | 132130 | *ycf1* | IRA | ACUM, DOLI, LISH, PSEU |
| 11 | P | 62174 | 71 | *accD-psaI* | LSC | 62174 | *accD-psaI* | LSC | ALL |
| 12 | F | 4733 | 77 | *trnK-UUU-rps16* | LSC | 109609 | *rrn23* | IRA | ALL |
| 13 | P | 4733 | 77 | *trnK-UUU-rps16* | LSC | 136426 | *rrn23* | IRB | ALL |
| 14 | F | 132036 | 62 | *ycf1* | IRB | 132150 | *ycf1* | IRB | ACUM, DOLI, LISH, PSEU |
| 15 | P | 54699 | 48 | *trnV-UAC-trnM-CAU* | LSC | 54699 | *trnV-UAC-trnM-CAU* | LSC | ALL |
| 16 | F | 113898 | 53 | *ycf1* | IRA | 113964 | *ycf1* | IRA | DOLI, LSIH, PSEU, KORE |
| 17 | P | 113898 | 53 | *ycf1* | IRA | 132095 | *ycf1* | IRB | DOLI, LISH, PSEU, KORE |
| 18 | P | 113964 | 53 | *ycf1* | IRA | 132161 | *ycf1* | IRB | DOLI, LISH, PSEU, KORE |
| 19 | F | 132095 | 53 | *ycf1* | IRB | 132161 | *ycf1* | IRB | DOLI, LISH, PSEU, KORE |
| 20 | F | 4761 | 49 | *trnK-UUU-rps16* | LSC | 109637 | *rrn23* | IRA | ACUM, DOLI, LISH, PSEU |
| 21 | P | 4761 | 49 | *trnK-UUU-rps16* | LSC | 136426 | *rrn23* | IRB | ACUM, DOLI, LISH, PSEU |
| 22 | P | 97913 | 49 | *trnL-CAA-ndhB* | IRA | 97913 | *trnL-CAA-ndhB* | IRA | ALL |
| 23 | F | 97913 | 49 | *trnL-CAA-ndhB* | IRA | 148150 | *ndhB-trnL-CAA* | IRB | ALL |
| 24 | P | 148150 | 49 | *ndhB-trnL-CAA* | IRB | 148150 | *ndhB-trnL-CAA* | IRB | ALL |
| 25 | P | 29927 | 44 | *petN-psbM* | LSC | 29927 | *petN-psbM* | LSC | ALL |
| 26 | F | 74280 | 50 | *clpP_extron_2-clpP_extron_1* | LSC | 74328 | *clpP_extron_2-clpP_extron_1* | LSC | ALL |
| 27 | F | 113971 | 46 | *ycf1* | IRA | 114019 | *ycf1* | IRA | DOLI, LISH, PSEU, KORE |
| 28 | P | 113971 | 46 | *ycf1* | IRA | 132047 | *ycf1* | IRB | DOLI, LISH, PSEU, KORE |
| 29 | P | 114019 | 46 | *ycf1* | IRA | 132095 | *ycf1* | IRB | DOLI, LISH, PSEU, KORE |
| 30 | F | 132047 | 46 | *ycf1* | IRB | 132095 | *ycf1* | IRB | DOLI, LISH, PSEU, KORE |
| 31 | F | 91981 | 37 | *ycf2* | IRA | 92038 | *ycf2* | IRA | DOLI, LISH, PSEU, KORE |
| 32 | P | 91981 | 37 | *ycf2* | IRA | 154037 | *ycf2* | IRB | DOLI, LISH, PSEU, KORE |
| 33 | P | 92038 | 37 | *ycf2* | IRA | 154094 | *ycf2* | IRB | DOLI, LISH, PSEU, KORE |
| 34 | F | 154037 | 37 | *ycf2* | IRB | 154094 | *ycf2* | IRB | DOLI, LISH, PSEU, KORE |
| 35 | F | 132108 | 40 | *ycf1* | IRB | 132174 | *ycf1* | IRB | DOLI, LISH, PSEU, KORE |
| 36 | F | 8044 | 36 | *psbK-psbI* | LSC | 8080 | *psbK-psbI* | LSC | LISH |
| 37 | P | 5912 | 35 | *trnK-UUU-rps16, rps16* | LSC | 103495 | *trnV-GAC-rrn16* | IRA | ACUM, DOLI, LISH, PSEU |
| 38 | F | 5912 | 35 | *trnK-UUU-rps16, rps16* | LSC | 142582 | *rrn16-trnV-GAC* | IRB | ACUM, DOLI, LISH, PSEU |
| 39 | P | 65690 | 35 | *petA-psbJ* | LSC | 65732 | *petA-psbJ* | LSC | ACUM, DOLI, LISH, PSEU |
| 40 | P | 49642 | 43 | *trnL-UAA-trnF-GAA* | LSC | 49642 | *trnL-UAA-trnF-GAA* | LSC | ACUM, DOLI, LISH, PSEU |
| 41 | F | 113816 | 43 | *ycf1* | IRA | 113828 | *ycf1* | IRA | ALL |
| 42 | P | 113816 | 43 | *ycf1* | IRA | 132241 | *ycf1* | IRB | ALL |
| 43 | P | 113828 | 43 | *ycf1* | IRA | 132253 | *ycf1* | IRB | ALL |
| 44 | F | 132241 | 43 | *ycf1* | IRB | 132253 | *ycf1* | IRB | ACUM, DOLI, LISH, PSEU |
| 45 | F | 132060 | 33 | *ycf1* | IRB | 132108 | *ycf1* | IRB | DOLI, LISH, PSEU |
| 46 | F | 44907 | 39 | *ycf3_extron_2-ycf3_extron_1* | LSC | 101936 | *rps12-trnV-GAC* | IRA | ALL |
| 47 | P | 44907 | 39 | *ycf3_extron_2-ycf3_extron_1* | LSC | 144137 | *trnV-GAC-rps12* | IRB | ALL |
| 48 | P | 8621 | 36 | *psbI-trnS-GCU, trnS-GCU* | LSC | 46643 | *trnS-GGA, trnS-GGA-rps4* | LSC | ACUM, DOLI, LISH, PSEU |
| 49 | F | 5491 | 32 | *trnK-UUU-rps16* | LSC | 5642 | *trnK-UUU-rps16* | LSC | DOLI, LISH |
| PSEU |  |  |  |  |  |  |  |  |  |
| 1 | P | 5829 | 131 | *trnK-UUU-rps16, rps16* | LSC | 103594 | *trnV-GAC-rrn16, rrn16* | IRA | ACUM, DOLI, LISH, PSEU |
| 2 | F | 5829 | 131 | *trnK-UUU-rps16, rps16* | LSC | 141974 | *rrn16, rrn16-trnV-GAC* | IRB | ACUM, DOLI, LISH, PSEU |
| 3 | F | 4637 | 107 | *trnK-UUU-rps16* | LSC | 109596 | *rrn23* | IRA | ALL |
| 4 | P | 4637 | 107 | *trnK-UUU-rps16* | LSC | 135996 | *rrn23* | IRB | ALL |
| 5 | F | 113999 | 82 | *ycf1* | IRA | 114113 | *ycf1* | IRA | ACUM, DOLI, LISH, PSEU |
| 6 | P | 113999 | 82 | *ycf1* | IRA | 131504 | *ycf1* | IRA | ACUM, DOLI, LISH, PSEU |
| 7 | P | 114113 | 82 | *ycf1* | IRA | 131618 | *ycf1* | IRA | ACUM, DOLI, LISH, PSEU |
| 8 | F | 131504 | 82 | *ycf1* | IRA | 131618 | *ycf1* | IRA | ACUM, DOLI, LISH, PSEU |
| 9 | P | 62163 | 71 | *accD-psaI* | LSC | 62163 | *accD-psaI* | LSC | ALL |
| 10 | F | 4747 | 77 | *trnK-UUU-rps16* | LSC | 109708 | *rrn23* | IRA | ALL |
| 11 | P | 4747 | 77 | *trnK-UUU-rps16* | LSC | 135914 | *rrn23* | IRB | ALL |
| 12 | F | 131524 | 62 | *ycf1* | IRB | 131638 | *ycf1* | IRB | ACUM, DOLI, LISH, PSEU |
| 13 | F | 4691 | 53 | *trnK-UUU-rps16* | LSC | 109650 | *rrn23* | IRA | ACUM, DOLI, PSEU |
| 14 | P | 4691 | 53 | *trnK-UUU-rps16* | LSC | 135996 | *rrn23* | IRB | ACUM, DOLI, PSEU |
| 15 | P | 54718 | 48 | *trnV-UAC-trnM-CAU* | LSC | 54718 | *trnV-UAC-trnM-CAU* | LSC | ALL |
| 16 | F | 113997 | 53 | *ycf1* | IRA | 114063 | *ycf1* | IRA | DOLI, LISH, PSEU, KORE |
| 17 | P | 113997 | 53 | *ycf1* | IRA | 131583 | *ycf1* | IRB | DOLI, LISH, PSEU, KORE |
| 18 | P | 114063 | 53 | *ycf1* | IRA | 131649 | *ycf1* | IRB | DOLI, LISH, PSEU, KORE |
| 19 | F | 131583 | 53 | *ycf1* | IRB | 131649 | *ycf1* | IRB | DOLI, LISH, PSEU, KORE |
| 20 | F | 4775 | 49 | *trnK-UUU-rps16* | LSC | 109736 | *rrn23* | IRA | ACUM, DOLI, LISH, PSEU |
| 21 | P | 4775 | 49 | *trnK-UUU-rps16* | LSC | 135914 | *rrn23* | IRB | ACUM, DOLI, LISH, PSEU |
| 22 | P | 98018 | 49 | *trnL-CAA-ndhB* | IRA | 98018 | *trnL-CAA-ndhB* | IRA | ALL |
| 23 | F | 98018 | 49 | *trnL-CAA-ndhB* | IRA | 147632 | *ndhB-trnL-CAA* | IRB | ALL |
| 24 | P | 147632 | 49 | *ndhB-trnL-CAA* | IRB | 147632 | *ndhB-trnL-CAA* | IRB | ALL |
| 25 | P | 29962 | 44 | *petN-psbM* | LSC | 29962 | *petN-psbM* | LSC | ALL |
| 26 | F | 72184 | 47 | *rps12-clpP* | LSC | 72229 | *rps12-clpP* | LSC | PPSEU |
| 27 | F | 74376 | 50 | *clpP_extron_2-clpP_extron_1* | LSC | 74424 | *clpP_extron_2-clpP_extron_1* | LSC | ALL |
| 28 | F | 114070 | 46 | *ycf1* | IRA | 114118 | *ycf1* | IRA | DOLI, LISH, PSEU, KORE |
| 29 | P | 114070 | 46 | *ycf1* | IRA | 131535 | *ycf1* | IRB | DOLI, LISH, PSEU, KORE |
| 30 | P | 114118 | 46 | *ycf1* | IRA | 131583 | *ycf1* | IRB | DOLI, LISH, PSEU, KORE |
| 31 | F | 131535 | 46 | *ycf1* | IRB | 131583 | *ycf1* | IRB | DOLI, LISH, PSEU, KORE |
| 32 | F | 92092 | 37 | *ycf2* | IRA | 92149 | *ycf2* | IRA | DOLI, LISH, PSEU, KORE |
| 33 | P | 92092 | 37 | *ycf2* | IRA | 153513 | *ycf2* | IRB | DOLI, LISH, PSEU, KORE |
| 34 | P | 92149 | 37 | *ycf2* | IRA | 153570 | *ycf2* | IRB | DOLI, LISH, PSEU, KORE |
| 35 | F | 153513 | 37 | *ycf2* | IRB | 153570 | *ycf2* | IRB | DOLI, LISH, PSEU, KORE |
| 36 | F | 131596 | 40 | *ycf1* | IRB | 131662 | *ycf1* | IRB | DEWU, LISH, PSEU,KORE |
| 37 | P | 5925 | 35 | *trnK-UUU-rps16, rps16* | LSC | 103594 | *trnV-GAC-rrn16* | IRA | ACUM, DOLI, LISH, PSEU |
| 38 | F | 5925 | 35 | *trnK-UUU-rps16, rps16* | LSC | 142070 | *rrn16-trnV-GAC* | IRB | ACUM, DOLI, LISH, PSEU |
| 39 | P | 65689 | 35 | *petA-psbJ* | LSC | 65731 | *petA-psbJ* | LSC | ACUM, DOLI, LISH, PSEU |
| 40 | P | 49663 | 43 | *trnL-UAA-trnF-GAA* | LSC | 49663 | *trnL-UAA-trnF-GAA* | LSC | ACUM, DOLI, LISH, PSEU |
| 41 | F | 113915 | 43 | *ycf1* | IRA | 113927 | *ycf1* | IRA | ALL |
| 42 | P | 113915 | 43 | *ycf1* | IRA | 131729 | *ycf1* | IRB | ALL |
| 43 | P | 113927 | 43 | *ycf1* | IRA | 131741 | *ycf1* | IRB | ALL |
| 44 | F | 131729 | 43 | *ycf1* | IRB | 131741 | *ycf1* | IRB | ACUM, DOLI, LISH, PSEU |
| 45 | F | 131548 | 33 | *ycf1* | IRB | 131596 | *ycf1* | IRB | DOLI, LISH, PSEU |
| 46 | F | 44929 | 39 | *ycf3_extron_2-ycf3_extron_1* | LSC | 102035 | *rps12-trnV-GAC* | IRA | ALL |
| 47 | P | 44929 | 39 | *ycf3_extron_2-ycf3_extron_1* | LSC | 143625 | *trnV-GAC-rps12* | IRB | ALL |
| 48 | P | 8647 | 36 | *psbI-trnS-GCU, trnS-GCU* | LSC | 46665 | *trnS-GGA, trnS-GGA-rps4* | LSC | ACUM, DOLI, LISH, PSEU |
| 49 | P | 62081 | 31 | *accD-psaI* | LSC | 62119 | *accD-psaI* | LSC | PSEU |
| KORE |  |  |  |  |  |  |  |  |  |
| 1 | F | 4650 | 107 | *trnK-UUU-rps16* | LSC | 110141 | *rrn23* | IRA | ALL |
| 2 | P | 4650 | 107 | *trnK-UUU-rps16* | LSC | 136530 | *rrn23* | IRB | ALL |
| 3 | F | 7459 | 91 | *trnQ-UUG-psbK* | LSC | 7546 | *trnQ-UUG-psbK* | LSC | KORE |
| 4 | P | 5839 | 95 | *rps16_extron_2* | LSC | 104175 | *trnV-GAC-rrn16, rrn16* | IRA | KORE |
| 5 | F | 5839 | 95 | *rps16_extron_2* | LSC | 142508 | *rrn16, rrn16-trnV-GAC* | IRB | KORE |
| 6 | F | 4760 | 77 | *trnK-UUU-rps16* | LSC | 110253 | *rrn23* | IRA | ALL |
| 7 | P | 4760 | 77 | *trnK-UUU-rps16* | LSC | 136448 | *rrn23* | IRB | ALL |
| 8 | P | 62607 | 71 | *accD-psaI* | LSC | 62607 | *accD-psaI* | LSC | ALL |
| 9 | P | 5869 | 65 | *rps16_extron_2* | LSC | 104175 | *trnV-GAC-rrn16, rrn16* | IRA | kORE |
| 10 | F | 5869 | 65 | *rps16_extron_2* | LSC | 142538 | *rrn16, rrn16-trnV-GAC* | IRB | KORE |
| 11 | P | 5934 | 57 | *rps16_extron_2* | LSC | 104120 | *trnV-GAC-rrn16, rrn16* | IRA | KORE |
| 12 | F | 5934 | 57 | *rps16_extron_2* | LSC | 142601 | *rrn16, rrn16-trnV-GAC* | IRB | KORE |
| 13 | P | 55130 | 48 | *trnV-UAC-trnM-CAU* | LSC | 55130 | *trnV-UAC-trnM-CAU* | LSC | ALL |
| 14 | F | 114517 | 53 | *ycf1* | IRA | 114584 | *ycf1* | IRA | DOLI, LISH, PSEU, KORE |
| 15 | P | 114517 | 53 | *ycf1* | IRA | 132141 | *ycf1* | IRB | DOLI, LISH, PSEU, KORE |
| 16 | P | 114583 | 53 | *ycf1* | IRA | 132207 | *ycf1* | IRB | DOLI, LISH, PSEU, KORE |
| 17 | F | 132141 | 53 | *ycf1* | IRB | 132207 | *ycf1* | IRB | DOLI, LISH, PSEU, KORE |
| 18 | P | 98563 | 49 | *trnL-CAA-ndhB* | IRA | 98563 | *trnL-CAA-ndhB* | IRA | ALL |
| 19 | F | 98563 | 49 | *trnL-CAA-ndhB* | IRA | 148166 | *ndhB-trnL-CAA* | IRB | ALL |
| 20 | P | 148166 | 49 | *ndhB-trnL-CAA* | IRB | 148166 | *ndhB-trnL-CAA* | IRB | ALL |
| 21 | F | 114540 | 55 | *ycf1* | IRA | 114655 | *ycf1* | IRA | KORE |
| 22 | P | 114540 | 55 | *ycf1* | IRA | 132068 | *ycf1* | IRB | KORE |
| 23 | P | 114654 | 55 | *ycf1* | IRA | 132182 | *ycf1* | IRB | KORE |
| 24 | F | 132068 | 55 | *ycf1* | IRB | 132182 | *ycf1* | IRB | KORE |
| 25 | P | 30349 | 44 | *petN-psbM* | LSC | 30349 | *petN-psbM* | LSC | ALL |
| 26 | F | 74866 | 50 | *clpP_extron_2-clpP_extron_1* | LSC | 74914 | *clpP_extron_2-clpP_extron_1* | LSC | ALL |
| 27 | F | 114532 | 49 | *ycf1* | IRA | 114647 | *ycf1* | IRA | KORE |
| 28 | P | 114532 | 49 | *ycf1* | IRA | 132082 | *ycf1* | IRB | KORE |
| 29 | P | 114646 | 49 | *ycf1* | IRA | 132196 | *ycf1* | IRB | KORE |
| 30 | F | 132082 | 49 | *ycf1* | IRB | 132196 | *ycf1* | IRB | KORE |
| 31 | F | 92631 | 37 | *ycf2* | IRA | 92688 | *ycf2* | IRA | DOLI, LISH, PSEU, KORE |
| 32 | P | 92631 | 37 | *ycf2* | IRA | 154053 | *ycf2* | IRB | DOLI, LISH, PSEU, KORE |
| 33 | P | 92688 | 37 | *ycf2* | IRA | 154110 | *ycf2* | IRB | DOLI, LISH, PSEU, KORE |
| 34 | F | 154053 | 37 | *ycf2* | IRB | 154110 | *ycf2* | IRB | DOLI, LISH, PSEU, KORE |
| 35 | F | 114590 | 46 | *ycf1* | IRA | 114639 | *ycf1* | IRA | DOLI, LISH, PSEU, KORE |
| 36 | P | 114590 | 46 | *ycf1* | IRA | 132093 | *ycf1* | IRB | DOLI, LISH, PSEU, KORE |
| 37 | P | 114638 | 46 | *ycf1* | IRA | 132141 | *ycf1* | IRB | DOLI, LISH, PSEU, KORE |
| 38 | F | 132093 | 46 | *ycf1* | IRB | 132141 | *ycf1* | IRB | DOLI, LISH, PSEU, KORE |
| 39 | F | 132154 | 40 | *ycf1* | IRB | 132220 | *ycf1* | IRB | DOLI, LISH, PSEU, KORE |
| 40 | P | 66136 | 34 | *petA-psbJ* | LSC | 66181 | *petA-psbJ* | LSC | KORE |
| 41 | F | 114435 | 43 | *ycf1* | IRA | 114448 | *ycf1* | IRA | ALL |
| 42 | P | 114435 | 43 | *ycf1* | IRA | 132287 | *ycf1* | IRB | ALL |
| 43 | P | 114447 | 43 | *ycf1* | IRA | 132299 | *ycf1* | IRB | ALL |
| 44 | F | 114558 | 43 | *ycf1* | IRB | 114673 | *ycf1* | IRB | KORE |
| 45 | P | 114558 | 43 | *ycf1* | SSC | 132062 | *ycf1* | IRB | KORE |
| 46 | P | 114672 | 43 | *ycf1* | SSC | 132176 | *ycf1* | IRB | KORE |
| 47 | F | 132287 | 43 | *ycf1* | IRB | 132299 | *ycf1* | IRB | KORE |
| 48 | F | 45323 | 39 | *ycf3_extron_2-ycf3_extron_1* | LSC | 102580 | *rps12-trnV-GAC* | IRA | ALL |
| 49 | P | 45323 | 39 | *ycf3_extron_2-ycf3_extron_1* | LSC | 144159 | *trnV-GAC-rps12* | IRB | ALL |
